# Histone deacetylase (HDAC) 1 and 2 complexes regulate both histone acetylation and crotonylation *in vivo*

**DOI:** 10.1038/s41598-018-32927-9

**Published:** 2018-10-02

**Authors:** R. D. W. Kelly, A. Chandru, P. J. Watson, Y. Song, M. Blades, N. S. Robertson, A. G. Jamieson, J. W. R. Schwabe, S. M. Cowley

**Affiliations:** 1 Department of Molecular and Cell biology, Henry Wellcome Building, University of Leicester, Leicester, LE1 7RH UK; 2 Institute of Structural and Chemical biology, Henry Wellcome Building, Department of Molecular and Cell biology, University of Leicester, Leicester, LE1 7RH UK; 3 Bioinformatics and Biostatistics Analysis Support Hub (B/BASH), University of Leicester, Leicester, LE1 7RH UK; 4 School of Chemistry, Joseph Black Building, University Avenue, University of Glasgow, Glasgow, G12 8QQ Scotland; 5 Department of Chemistry, University of Cambridge, Cambridge, CB2 1GA UK

## Abstract

Proteomic analysis of histones has shown that they are subject to a superabundance of acylations, which extend far beyond acetylation, to include: crotonylation, propionylation, butyrylation, malonylation, succinylation, β-hydroxybutyrylation and 2-hydroxyisobutyrylation. To date, much of the functional data has focussed on histone crotonylation which, similar to acetylation, has been associated with positive gene regulation and is added by the acyltransferase, p300. Although Sirtuins 1–3, along with HDAC3, have been shown to possess decrotonylase activity *in vitro*, there is relatively little known about the regulation of histone crotonylation *in vivo*. Here we show that Histone Deacetylase 1 and 2 (HDAC1/2), the catalytic core of numerous co-repressor complexes, are important histone decrotonylase enzymes. A ternary complex of HDAC1/CoREST1/LSD1 is able to hydrolyse both histone H3 Lys18-acetyl (H3K18ac) and H3 Lys18-crotonyl (H3K18cr) peptide substrates. Genetic deletion of HDAC1/2 in ES cells increases global levels of histone crotonylation and causes an 85% reduction in total decrotonylase activity. Furthermore, we mapped H3K18cr in cells using ChIP-seq, with and without HDAC1/2, and observed increased levels of crotonylation, which largely overlaps with H3K18ac in the vicinity of transcriptional start sites. Collectively, our data indicate that HDAC1/2 containing complexes are critical regulators of histone crotonylation *in vivo*.

## Introduction

Histone post-translational modifications (PTM) modulate gene expression and chromatin structure in all eukaryotic cells^1^. The most widely studied PTM, histone acetylation, neutralizes positively charged lysine residues in histone tails, decreasing nucleosomal affinity for DNA and is therefore associated with transcriptionally active chromatin^2^. Mass spectrometry-based proteomics has recently expanded the repertoire of different histone ‘acylations’ far beyond acetylation to include: crotonylation^3^ propionylation^4^, butyrylation^4^, malonylation^5^, succinylation^5,6^, β-hydroxybutyrylation^7^ and 2-hydroxyisobutyrylation^8^. These newly characterised histone PTMs have increased the complexity of chromatin biology and raise questions regarding how this assortment of histone acylations influence chromatin structure and function^6^. Furthermore, the proteins/factors responsible for the addition (writer), recognition (reader) and removal (erasure) of these acyl-lysine modifications remain poorly understood.

A number of functional studies have concentrated on histone lysine-crotonylation (Lys-cr) which is associated with active chromatin and overlaps with known sites of lysine-acetylation (Lys-ac)^3,9^. Both Lys-ac and Lys-cr share a common ‘writer’ in the acyltransferase and co-activator protein, p300^9^. However, Lys-ac and Lys-cr may be interpreted differently, since they are recognized by distinct readers, in the bromodomain^10^ and Yeats domain^11^ respectively. Although both PTM’s utilize p300, Lys-ac is by far the more abundant modification due to the high levels of acetyl-coA in relation to crotonyl-coA^9^. p300 also displays a preferences for short-chain acylations with reactivity decreasing proportionally to chain length^12^. The abundance of all histone PTMs is balanced through the opposing activities of writers and erasers. Since p300 functions as the common acyl-transferase, by extension it seemed reasonable that known histone deacetylases (HDACs) may have decrotonylase activity. Mammalian cells contain 18 HDAC enzymes that can be sub-divided into four main classes: class-I (HDAC1, 2, 3 and 8), class-II (HDAC4, 5, 6, 7, 9, 10), class-III (sirtuins 1–7) and class-IV (HDAC11) (reviewed by^13,14^). While sirtuins 1–3 have robust deacetylase activity^14^, the sirtuin family on the whole have a preference for substrates with an extended carbon-chain, including, succinyl and malonyl^15^ moieties and long-chain fatty acyl groups such as decanoyl and dodecanoyl^16^. Unsurprisingly, given the range of their potential substrates, sirtuins 1, 2^16^ and 3^17^ were shown to be decrotonylases *in vitro*. The zinc-dependent HDACs (1–11) are catalytically distinct from sirtuins (which utilize NAD+). A study profiling substrate preferences of HDAC1–11 identified HDAC3 as a potential decrotonylase^18^. However, these studies were performed *in vitro* with recombinant enzymes produced in isolation and therefore do not fully address the physiological role of Lys-cr erasers in a cellular context.

HDAC1 and HDAC2 (HDAC1/2) are highly related deacetylases that form the catalytic core of multiple co-repressor complexes, including, Sin3, NuRD, CoREST and MiDAC^19,20^. The complex is an essential component for HDAC1/2 activity, since they both activate their enzymatic activity and recruit them to their cellular targets. Recently, HDAC1/2 have been implicated in the regulation of histone crotonylation levels in cells^21,22^. siRNA knockdown of HDAC1/2^21^ or treatment with HDAC inhibitors^22^ caused increased levels of histone crotonylation; although counterintuitively, the average level of H3K18cr at transcriptional start sites (TSS) was reduced following HDAC inhibitor treatment^22^. Here we extend these data by showing a purified ternary complex of HDAC1/CoREST1/LSD1 is able to directly hydrolyse both histone H3 Lys18-acetyl (H3K18ac) and H3 Lys18-crotonyl (H3K18cr) peptide substrates. Genetic deletion of HDAC1/2 in embryonic stem (ES) cells increases global levels of histone crotonylation and caused an 85% reduction in total decrotonylase activity. Furthermore, we mapped global loci of H3K18ac and H3K18cr using ChIP-seq with and without HDAC1/2 in ES cells. In contrast to previously published data^22^, we observed increased levels of histone crotonylation upon loss of HDAC1/2 activity which largely overlaps with H3K18ac at TSS and correlates with gene activity. Collectively, these data indicate that HDAC1/2 containing complexes are critical regulators of histone crotonylation *in vivo*.

## Results

### HDAC1 functions as a histone decrotonylase whose activity is modulated by inositol phosphates

**Figure 1:**
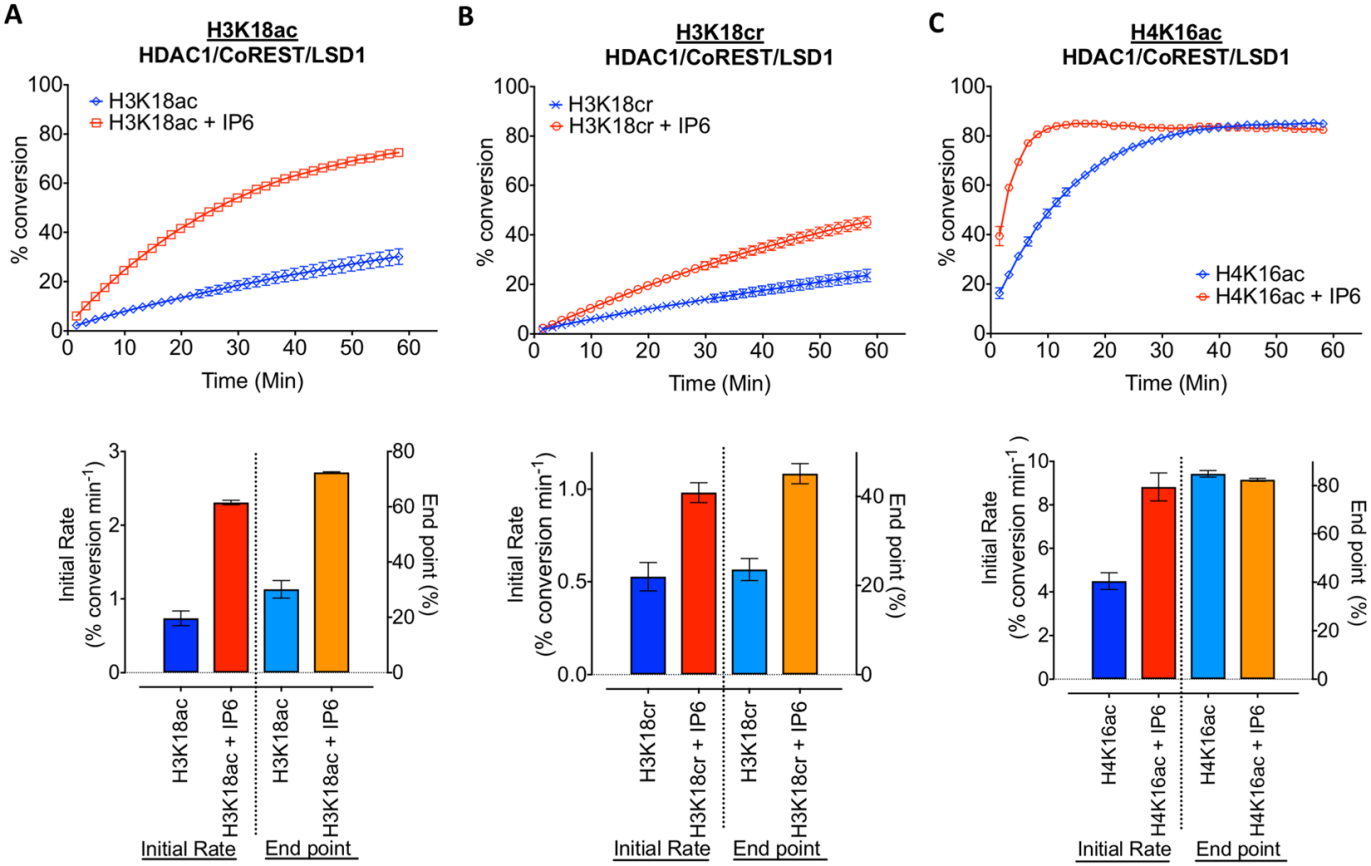
HDAC1 functions as a histone decrotonylase whose activity is modulated by inositol phosphates. Deacetylase and decrotonylase activities were measured using a Caliper assay with continuous substrate turnover for a 60 min period. Analogous H3K18ac (**A**), H3K18cr (**B**) and H4K16ac (**C**) fluorescein-tagged peptides were incubated with recombinant HDAC1/CoREST1/LSD1 ternary complex. Graphs show the average percentage conversion (mean ± SD; n = 2), initial rates and percentage conversion of substrate at the end point ±100 μM InsP_6_ (D-myo-inositol 1, 2, 3, 4, 5, 6-hexakisphosphate).

To investigate the putative decrotonylase activity of class-I HDACs we synthesized analogous histone H3 tail peptides containing H3K18ac and H3K18cr modifications (Supplementary Fig. 1A). H3K18 is the dominant site for both acetyltransferase and crotonyltransferase activity of p300^9^. In addition, we also generated the preferential substrate for class-I HDACs, H4K16Ac, as a positive control. HDAC1 is present in a number of co-repressor complexes, including, Sin3, NuRD and MiDAC^19,20^. For this study we utilized the CoREST complex because we are able to generate a recombinant HDAC1/CoREST1/LSD1 ternary complex^23^, using full-length proteins, in a 1:1:1 ratio at milligram quantities. Acetyl and crotonyl peptides were incubated with HDAC1/CoREST1/LSD1 for a 60 min period with continuous substrate turnover measured using a Caliper assay. HDAC1 was able to deacylate both H3K18ac and H3K18cr substrates (Fig. 1A,B), although the initial rate of decrotonylation was lower than deacetylation. At the end-point of the assay we were also able to measure the percentage conversion of each substrate, which was similarly reduced for H3K18cr compared to H3K18ac, suggesting that the crotonyl-lysine is turned over more slowly than the corresponding acetyl mark. Interestingly, both H3K18 modifications were hydrolysed at a reduced rate compared to H4K16Ac (Fig. 1C), indicating that there may be a hierarchy of acylated sites among histone tails. As a control we also tested the decrotonylase activity of a HDAC3/SMRT/GPS2/TBL1 quaternary complex. Consistent with previously published data^18^ HDAC3 also displayed significant activity against the H3K18cr peptide (Supplementary Fig. 1B), although similar to HDAC1, with an initial rate which was 2.4-fold lower than the H3K18ac substrate. The deacetylase activity of the HDAC1 and HDAC3 can be regulated by inositol phosphates (InsP)^23,24^, we therefore asked if InsP levels also help determine the rate of decrotonylation. The initial rate of deacetylation and decrotonylation was increased 3.2-fold and 1.8-fold respectively by the addition of InsP_6_ to the HDAC1/CoREST/LSD1 complex (Fig. 1A,B). Together these data demonstrate that both HDAC1 and HDAC3 containing complexes are active decrotonylases.

### HDAC inhibition in cells increases both H3K18ac and H3K18cr in a dose-dependent manner

**Figure 2:**
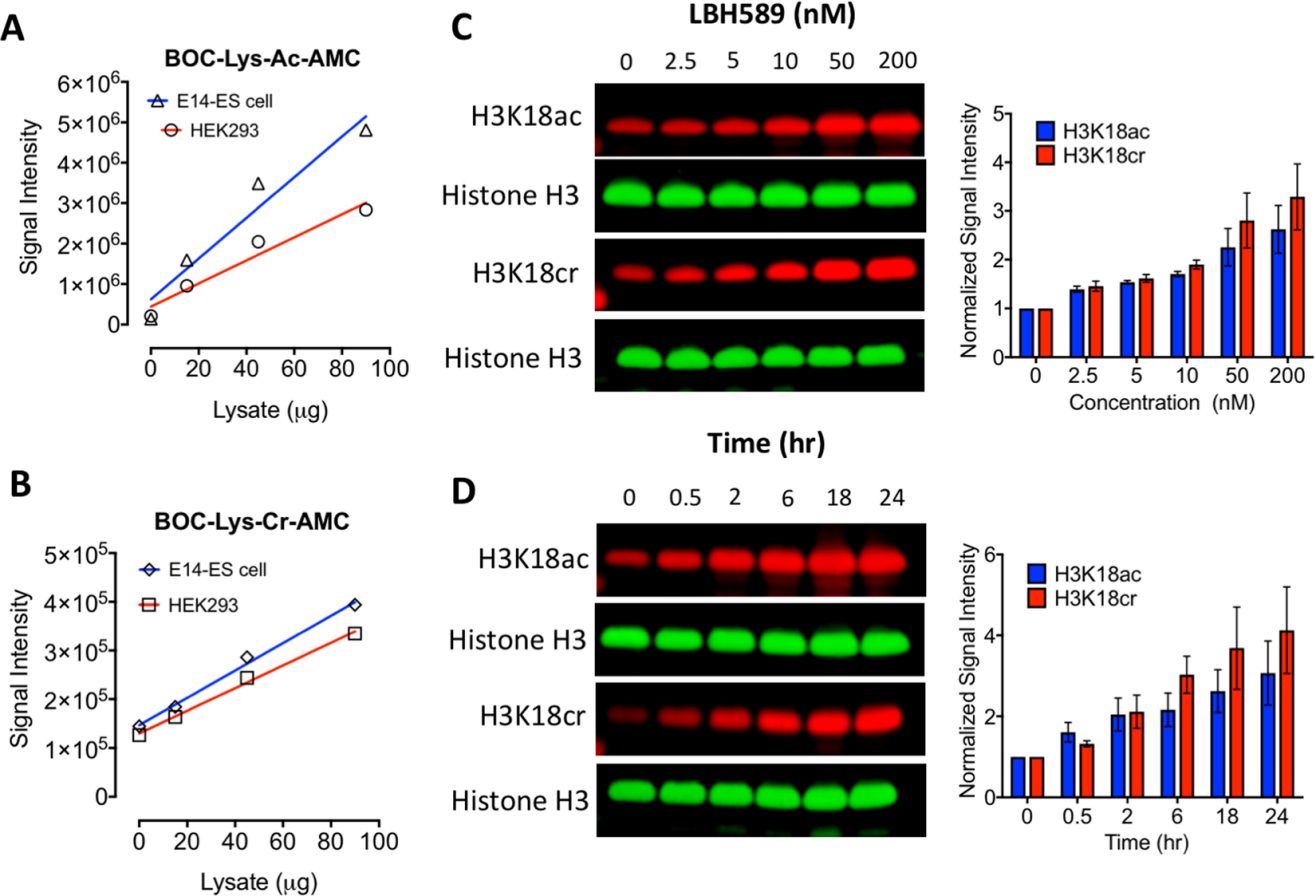
HDAC inhibition increases both H3K18ac and H3K18cr in a dose-dependent manner. (**A**) Deacetylase and (**B**) decrotonylase activities were measured using increasing concentrations of whole-cell extracts from mouse ES and HEK-293T cells using Boc-Lys(Ac)-AMC and BOC-Lys(Cr)-AMC substrates. Average plots of n = 3 technical replicates. ES cells were treated with either, increasing concentrations of LBH589 for 24 hrs (**C**), or treated with 50 nM of LBH589 for indicated time (**D**), before histones were extracted and subjected to quantitative western blotting using an Odyssey scanner. Levels of H3K18ac and H3K18cr were normalized to the level of histone H3 and graphs show the average normalized signal intensity (mean ± SEM; n ≥ 3). Uncropped scans of western blot gels are in Supplementary Fig. 2A,B.

To examine the decrotonylase activity in cells we generated a Boc-Lys(Cr)-AMC substrate, equivalent to the BOC-Lys(Ac)-AMC substrate used in our previous studies^25–27^. Initially, we used a standard 1-hour incubation period to measure deacetylase and decrotonylase activity but observed relatively little decrotonylase activity, even when using more than 90µg of whole cell extract (data not shown). Upon using extended incubation times (>6 hours) extracts from both ES cells and HEK-293T cells displayed robust decrotonylase activity which correlated with protein concentration (Fig. 2A,B). Treatment of ES cells with LBH589, a broad spectrum inhibitor of Zn^2+^-HDAC enzymes (HDACs 1–11) increased the levels of both H3K18cr (3.2-fold) and H3K18ac (2.6-fold) in a dose dependent manner (Fig. 2C). Although these are relatively modest increases, they are consistent with our previous data using ES cells, which display high basal levels of histone acetylation^25,28,29^. A time course of LBH589 treatment revealed that both H3K18ac and H3K18cr levels double within 2 hours (Fig. 2D), indicating that the dynamics of acetylation/crotonylation are similar in cells. To similarly address the kinetics of deacetylation/decrotonylation, cells were treated with LBH589 for 24 hours and then washed three times to remove the inhibitor, with the recovery time of H3K18ac and H3K18cr measured by western blotting. Surprisingly, given the difference in initial rates measured using the caliper assay *in vitro* (Fig. 1), we detect little or no difference in the recovery time, suggesting that the rate of decrotonylation is similar to deacetylation in cells (Supplementary Fig. S2C).

### HDAC1/2 are the dominant histone decrotonylases in ES cells

**Figure 3:**
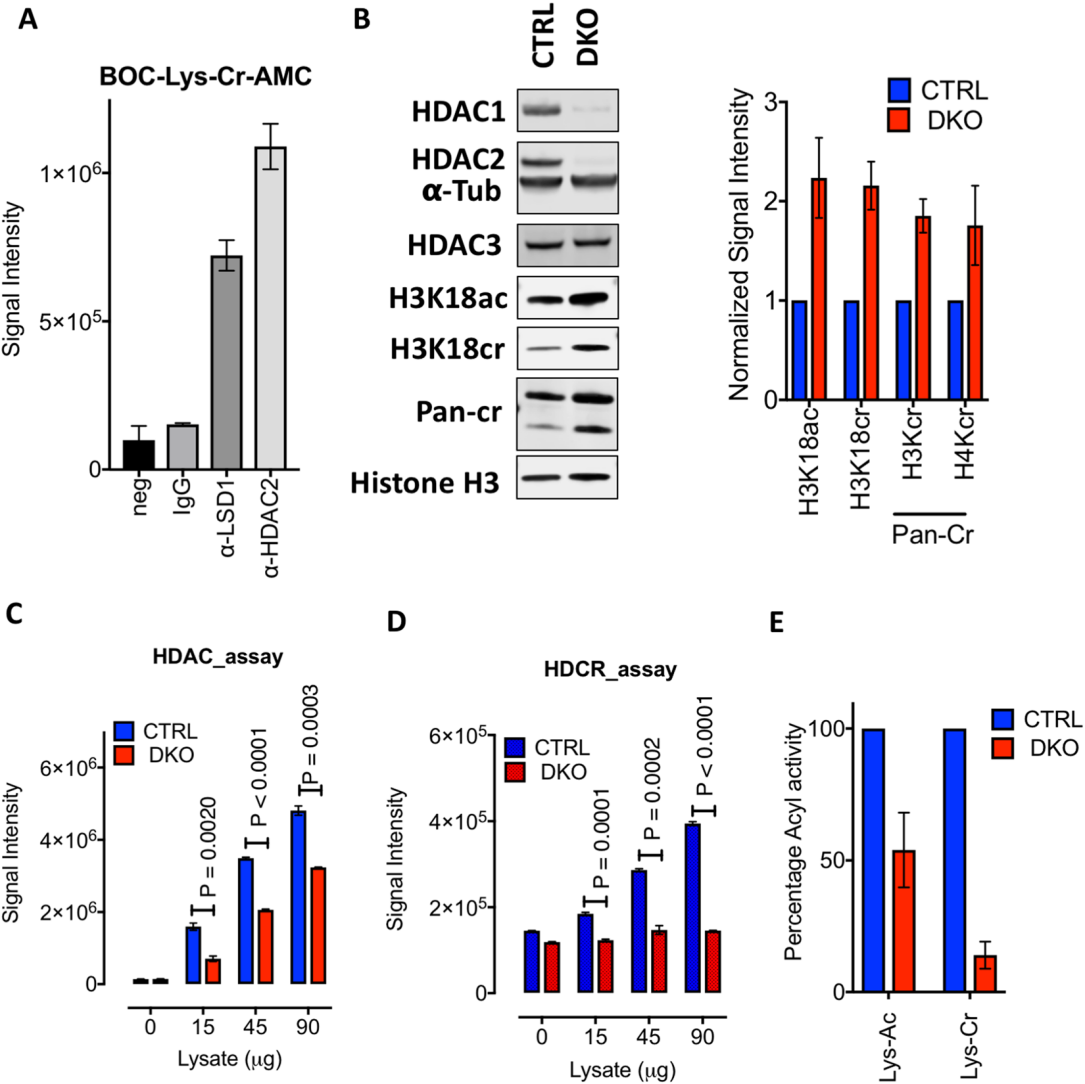
HDAC1/2 are the dominant histone decrotonylases in ES cells. (**A**) The decrotonylase activity of HDAC1/2 complexes isolated from ES cells, using antisera to HDAC2 and LSD1, were measured using a Boc-Lys(Cr)-AMC substrate. No cell lysate (neg) and normal mouse IgG were used as negative controls. Average (±SD) plots of n = 3 technical replicates. (**B**) Quantitative western blot data for the indicated proteins in Ctrl (day 0) and DKO ES cells treated with 4-hydroxytamoxifen (OHT) at day 3, were performed using an Odyssey scanner. Graphs show the average normalized signal intensity (mean ± SEM; n ≥ 3) relative to Histone H3. Uncropped scans of western blots are shown in Supplementary Fig. 3A–C. (**C**) Deacetylase and (**D**) decrotonylase assays were performed using whole-cell extracts from Ctrl (day 0) and DKO ES cells (day 3, following OHT treatment). Average (±SD) plots of n = 3 technical replicates. (**E**) Changes in both deacetylase and decrotonylase activity in DKO ESCs were calculated as a percentage of CTRL ES cells from the values depicted in (**C**) and (**D**).

Since ES cells contain a decrotonylase activity which is sensitive to LBH589 (Fig. 2C), a Zn^2+^-dependent HDAC inhibitor, we next asked if the highly related HDAC1 and HDAC2 (HDAC1/2) enzymes contributed to this activity. To isolate HDAC1/2 from cells, either directly or indirectly (as part of a higher-order complex), we used antisera to HDAC2 and LSD1, a key component of the CoREST complex, in a co-immunoprecipitation (co-IP) experiment. The level of decrotonylase activity in each co-IP was then measured using the Boc-Lys(Cr)-AMC substrate. Both HDAC2 and LSD1 related complexes demonstrated substantial decrotonylase activity (Fig. 3A). In many cell types HDAC1 and HDAC2 activity is redundant^26,30–32^, we have therefore generated ES cells in which both enzymes can be deleted conditionally^29^, hereafter referred to as double knockout (DKO) cells. Following deletion of HDAC1/2, quantitative western blotting revealed a 2.3-fold and 2.2-fold increase in H3K18ac and H3K18cr, respectively (Fig. 3B). Moreover, using a pan-crotonyl antibody, we were able to detect a 1.8-fold and 1.7-fold increase in total H3 and H4 crotonylation (Fig. 3B). Since these observations imply HDAC1/2 are active decrontonylases, we assayed the remaining deacetylase and decrotonylase activity following HDAC1/2 deletion. In the absence of HDAC1/2 we observed a significant decrease in both deacetylase (Fig. 3C) and decrotonylase (Fig. 3D) activity. There was a protein concentration dependent increase in HDAC activity even in the absence of HDAC1/2 (compare Ctrl v DKO), suggesting other HDACs were active within the extract (Fig. 3C). However, this was not observed for decrotonylase activity, where loss of HDAC1/2 resulted in little or no detectable decrotonylation activity (Fig. 3D). Indeed, decrotonylation activity was reduced by 85% compared to controls, while HDAC activity was only 56% of control levels (Fig. 3E). To assess whether loss of HDAC1/2 altered the expression of other decrotonylases, we examined HDAC3 protein levels in DKO cells, but found no change (Fig. 3B). In addition, we also examined H3K18ac and H3K18cr levels in HDAC3 knockout cells and found no significant changes (Supplementary Fig. 3B). The absence of a global change does not preclude HDAC3 regulating histone crotonylation at specific genomic loci. To further supplement these data we also tested histone crotonylation levels in compound HDAC1-KO; HDAC2-Het ES cells that have near normal levels of HDAC2 (Supplementary Fig. S3A), but overall reduced levels of deacetylase activity^29^. HDAC1-KO; HDAC2-Het cells showed increased levels of H3K18ac (1.5-fold) and histone H3 crotonylation (1.5-fold; Pan-Cr; Supplementary Fig. S2B), although these increases are smaller than those observed in DKO cells (Fig. 3B). This difference in activity suggests that HDAC2 acts as a histone decrotonylase, consistent with data from Wei *et al*.^21^ and that total decrotonylase activity (similar to deacetylase) correlates with the total amount of HDAC1/2. These results demonstrate that HDAC1/2 are the dominant decrotonylases in ES cells.

### HDAC1/2 regulates H3K18cr levels at active gene loci

**Figure 4:**
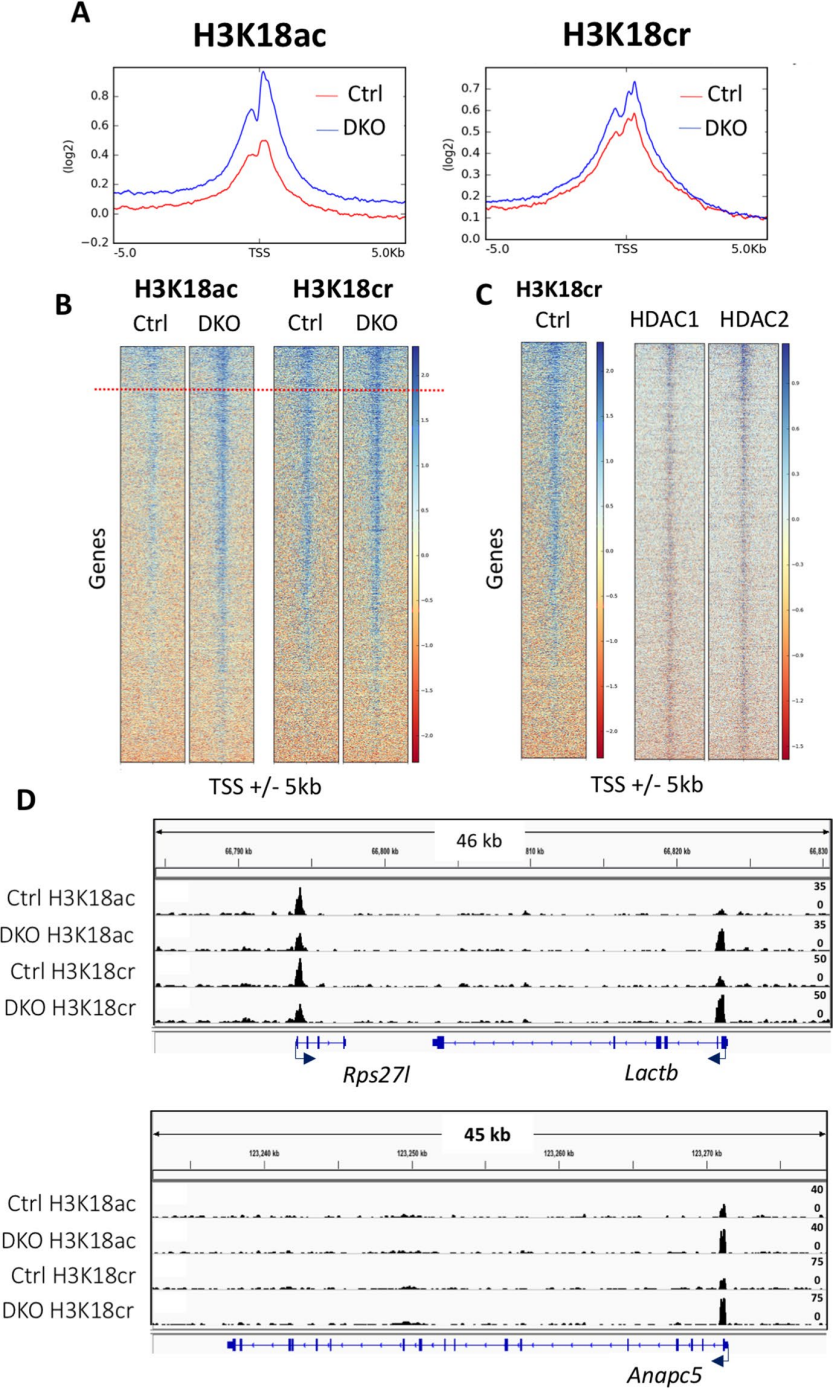
Loss of HDAC1/2 leads to a global increase in H3K18cr and H3K18ac. (**A**) Average ChIP coverage levels of H3K18ac and H3K18cr in Ctrl and DKO ES cells at transcriptional start sites (TSS) 5,000 bp upstream and downstream. (**B**) ChIP-seq density heatmaps in Ctrl and DKO ES cells for H3K18ac and H3K18cr at TSS ±5,000 bp, ranked by increase in acetylation or crotonylation following *Hdac1/2* deletion. Red dotted line indicates the top 10% of genes enriched for H3K18ac and H3K18cr. (**C**) Heatmap of HDAC1 and 2 densities [as defined in Whyte *et al*.^38^] at TSS sites ±5,000 bp ranked by H3K18cr levels in Ctrl cells. (**D**) IGV enrichment tracks of H3K18ac and H3K18cr at the three representative genes: *Rps27l*, *Lactb* and *Anapc5*. The gene structure and dimensions are indicated in each panel.

To investigate the regulation of histone crotonylation by HDAC1/2 at the genomic level, we performed chromatin immunoprecipitation followed by next generation sequencing (ChIP-Seq) for H3K18ac and H3K18cr in control and DKO ES cells. In line with previous findings^9^, we observed that H3K18cr levels peak in the vicinity of transcription start sites (TSS) with a significant overlap with H3K18ac (Fig. 4A). This is not surprising, since HDAC1/2 map predominantly to TSS (Fig. 4C) with a positive correlation between binding and gene activity^33,34^. Loss of HDAC1/2 increases the average levels of both H3K18ac and H3K18cr around the TSS (Ctrl vs DKO), with a more pronounced increase observed for H3K18ac. These elevated levels appear be largely at pre-existing sites of acetylation/crotonylation rather than *de novo* loci (Fig. 4B). At specific gene loci, such as *Lactb* or *Anapc5* (Fig. 4D), we observed increases in H3K18cr of up to 4-fold following HDAC1/2 deletion. These increases in histone crotonylation appear to be correlated with corresponding changes in H3K18ac, suggesting that HDAC1/2 co-ordinately regulate both histone modifications at specific sites.

**Figure 5:**
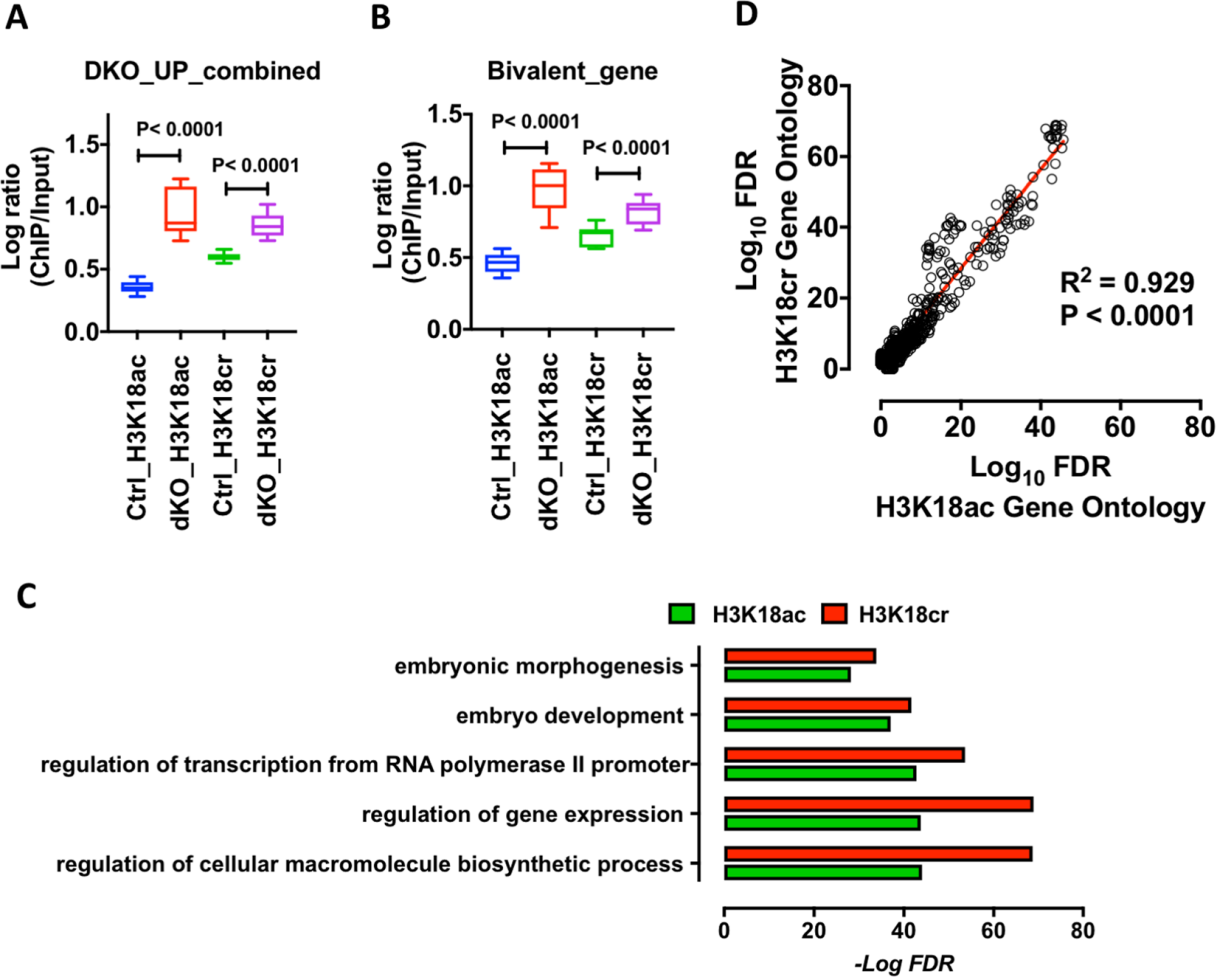
Loss of HDAC1/2 regulates H3K18cr levels associated with active gene expression. Average levels of H3K18ac and H3K18cr in Ctrl and DKO ES cells: (**A**) at upregulated genes following the loss of HDAC1/2 [as defined in Jamaladdin *et al*.^29^]; and (**B**) genes associated with bivalent histone signatures [as defined in Mikkelsen *et al*.^36^]. Acetylation level was calculated using DEEPTOOLS for each promoter, defined as the region between ±500 bp of the TSS, and presented in log_2_ scale. (**C**) Functional annotation analysis of the top 10% of genes enriched for either H3K18cr or H3K18ac (Fig. 4B; red line). DAVID was used to identify biological process (BP) and gene ontology (GO). (**D**) Regression analysis of false discovery rate (FDR) from enriched GO terms for both H3K18ac and H3K18cr. Data presented in log_10_ scale.

Given the association of H3K18ac with TSS, we next asked what effect deleting HDAC1/2 had on H3K18ac and H3K18cr levels at a specific subset of HDAC1/2 regulated genes. In a previous study, we monitored changes in the ES cell transcriptome following loss of HDAC1/2^29^. Cross-referencing the 1,445 genes up-regulated genes in DKO cells to our ChIP-seq dataset revealed a strong correlation between increased transcription and an increase in H3K18cr (Fig. 5A, p < 0.0001) and H3K18ac (p < 0.0001) levels. Individual genes, such as *Htra1*, *Hes6* and *Nagk*, whose expression increased 2.7, 1.6 and 2.1-fold respectively, showed corresponding increases in H3K18ac and H3K18cr levels (Supplementary Fig. S4). This demonstrates a concomitant relationship between HDAC1/2 gene regulation and histone crotonylation levels. Intriguingly, we also observed increased levels of both H3K18ac and H3K18cr at bivalent genes (Fig. 5B), that is, poised genes marked with both H3K4me3 and H3K27me3 in ES cells^35,36^, suggesting a role for HDAC1/2 in controlling the activity of developmentally regulated genes prior to ES cell differentiation. Consistent with this mechanism, gene ontology (GO) analysis of the top 10% of genes enriched for either H3K18cr or H3K18ac upon HDAC1/2 deletion (Fig. 4B; red line), revealed functional enrichment for genes with roles in embryonic morphogenesis and embryo development (Fig. 5C). Furthermore, by plotting the false discovery rate (FDR) of enriched GO terms for both H3K18ac and H3K18cr we observed an R^2^ = 0.929 correlation (p < 0.0001), indicating a significant overlap in the function of these two acylations in ES cells (Fig. 5D).

## Discussion

In this study, we show that HDAC1/2 and HDAC3 are active decrotonylase enzymes *in vitro* using recombinant purified co-repressor complexes (Fig. 1A and Supplementary Fig. 1B). Furthermore, genetic deletion of HDAC1/2 from ES cells reveals them to be critical enzymes in the regulation of H3K18cr levels, in terms of total decrotonylase activity (Fig. 3) and the regulation of histone crotonylation at specific genomic loci (Fig. 4). Wei *et al*.^21^ demonstrated that overexpression of class I HDACs (HDAC1, 2, 3 and 8) in HeLa cells were able to decrease global histone crotonylation levels, while knock-down or HDAC inhibitor treatment produced the opposite effect^21^. They also suggest that histone crotonylation may play a role in the pluripotency of ES cells. More recently, Fellows *et*. *al*. (2018) provided evidence that class-I HDACs regulate levels of histone decrotonylation in the intestine in response to short chain fatty acids generated by the microbiota of the gut^22^. Although treatment of HCT-116 cells with MS-275 increased the global levels of H3K18cr, ChIP-seq showed a reduction in H3K18cr levels around transcriptional start sites^22^. In contrast, we observed a small but significant increase in H3K18cr in the vicinity of TSS upon deletion of HDAC1/2 of in ES cells (Fig. 4A), which is more pronounced at specific promoters (*Lactb* and *Anapc5*, Fig. 4D). This is perhaps unsurprising since HDAC1/2 themselves (Fig. 4C) and their related complex components, Sin3A^37^ and LSD1^38^, all map within the vicinity of TSS. A comparison of H3K18ac and H3K18cr in ES cells revealed a significant overlap in loci, with the increase in abundance upon HDAC1/2 deletion occurring at pre-existing sites. We also observed an enrichment for H3K18cr at active genes, such *Nanog* and *Pou5f1* (Fig. 4B) consistent with a role for histone crotonylation in active gene expression^9,39^.

Previous studies failed to detect significant decrotonylase activity for HDAC1/2^3,18^ and this may be because they were using recombinant enzymes generated in isolation. The activity of class-I HDACs *in vivo* is dependent upon binding to an activating partner, part of a multi-protein complex^19,20^, as typified by HDAC1/CoREST/LSD1 and HDAC3/SMRT/GPS2/TBL1. A more physiological examination of class-I HDAC activity should therefore be performed as part of a complex. To that end, we show that an HDAC1/CoREST1/LSD1 ternary (Fig. 1) and HDAC3/SMRT/GPS2/TBL1 (Supplementary Fig. S1B) quaternary complex are able to turnover H3K18cr, albeit at a reduced rate compared to the equivalent acetylated substrate. This is consistent with data from Madsen and Olson^18^, who showed that the HDAC3/NCoR complex also hydrolysed Lys-cr at a lower rate than Lys-ac, although still comparable to the deacetylase activities of HDAC8 and HDAC11. Intriguingly, when we examined the rate of decrotonylation in cells (inhibitor treatment followed by wash-out and recovery, Supplementary Fig. S2C), we found there was little difference between deacetylation and decrotonylation rates. This may reflect the difference in substrates (isolated histone tails versus intact nucleosomes) or the presence of cooperating factors required for histone recognition. The Sin3A/HDAC1 complex for example, contains Ing1/2 (PhD finger) and ARID4A/B (tudor domain, chromodomain and AT-rich interacting domain) which assist recruitment of the complex to chromatin^19^.

Mass-spectrometry data has shown that there are an abundance of histone crotonylation sites in all four core histones^22,40^. To date, decrotonylase activity has been described for HDAC1-3 and Sirtuins 1-3^16–18,21,22^. It is entirely conceivable that each of these enzymes (and associated complexes) are important for regulating crotonylation at a subset of target genes or specific lysines within histone tails. We have shown that deletion of HDAC1/2 in ES cells caused an 85% reduction in total decrotonylase activity; this is the first genetic link between HDACs and decrotonylase activity in cells. Since HDAC1/2 can accommodate the unique double bond and hydrocarbon chain length of Lys-cr, it is reasonable to speculate that they may also depropionylate and debutyrylate histones, which have a chain length equal to or less than the crotonyl moiety^3,4^. Indeed, data from Fellows *et al*., showed that purified HDAC1, 2 and 3 are able to reduce the levels of H3K18 butyrlation *in vitro*^22^. Further examination of these modifications in cells within the context of class-I HDACs will be required, especially since crotonylation^9^, propionylation and butyrylation are all associated with active chromatin^41,42^. Exploring the physiological requirement for the galaxy of newly described histone acylations, their ‘readers’ such as the Yeats domain^11^, and the enzymes involved in their deposition and erasure will no doubt increase our understanding of chromatin biology and HDAC function.

## Methods

### Cell culture

Murine embryonic stem cells were cultured on gelatinized plates in media composed of Knock-Out Dulbecco’s Modified Eagle Media (DMEM), supplemented with 15% fetal bovine serum (FBS), 1 μg/mL Streptomycin, 1 unit/mL Penicillin, 2 mM L-glutamine, 0.1 mM β-mercaptoethanol and recombinant leukemia inhibitory factor (LIF) in a humidified incubator at 37 °C and 5% CO_2_. To induce the deletion of *Hdac1/2*, DKO ES cells were cultured with 1 μM tamoxifen (OHT) for 24 h to activate the CreER fusion protein. HEK-293T cells were cultured in DMEM supplemented with 10% FBS, 1 μg/mL, Streptomycin 1 unit/mL Penicillin, 2 mM L-glutamine at in a humidified incubator at 37 °C and 5% CO_2_. Suspension cultures of HEK-293F cells were cultured in 250 ml conical tissue culture flasks (60 ml cultures) in FreeStyle^TM^ HEK-293F Expression Medium in a humidified shaking incubator (120 rpm) at 37 °C and 5% CO_2_

### *In vitro* Histone Deacetylation (HDAC) and decrotonylation (HDCR) Assays

The transfection, expression and purification of HDAC3/SMRT/TBL1/GPS2 quaternary and HDAC1/CoREST1/LSD1 ternary complexes were performed as previously described^23,43^. Real time HDAC and HDCR activity of purified HDAC complexes were determined using the Caliper EZ Reader II system (Caliper Life Sciences, www.caliperls.com) as published previously (46), using 500 nM HDAC1/CoREST1/LSD1 or 500 nM HDAC3/SMRT/GPS2/TBL1 with fluorescein-labelled H3K18ac, H3K18cr and H4K16Ac peptide substrates. A detailed description of the synthesis and characterization of acetylated and crotonylated peptides can be found in the Supplemental Methods. To assess the effect of inositol phosphate, the HDAC1/CoREST/LSD1 ternary complex was pre-mixed with or without InsP_6_ (D-myo-inositol 1,2,3,4,5,6-hexakisphosphate) before dilution and addition of substrate. All reactions were carried out in duplicate in 30 µl reaction volumes, performed at room temperature in 50 mM Tris pH 7.5, 50 mM NaCl, 5% glycerol. Data was analysed using GraphPad Prism (version 6.0).

For HDAC and HDCR assays using Boc-Lys(Ac)-AMC and Boc-Lys(Cr)-AMC substrates, cells were extracted in HDAC assay buffer containing 50 mM NaCl, 50 mM Tris-HCL pH 7.5, 5% Glycerol, 0.3% Triton X-100 at 4 °C for 20 min. Samples were cleared at 20,000 × *rcf* for 15 min. A Bradford assay was conducted to estimate the total protein concentration. Between 0–90 μg of protein extract was mixed and incubated with 100 μM BOC-Lys(Ac)-AMC (Sigma-Aldrich, UK) or 100 μM BOC-Lys(Cr)-AMC in HDAC buffer for 1 hr for the HDAC assay or 18 hr for the HDCR assay. The reaction was stopped and developed using 2 mM TSA and 10 mg/ml Trypsin diluted in HDAC assay buffer. Fluorescence was measured at 335/460 nm using a Victor X5 plate reader (Perkin Elmer). A detailed description of synthesis and characterization of Boc-Lys(Cr)-AMC substrate can be found in the Supplemental Methods.

### Protein extraction and Western Blotting

Whole-cell extracts were prepared in immunoprecipitation (IP) buffer (150 mM NaCl, 50 mM Tris-HCL pH 7.5, 0.5% IEGPAL and protease inhibitor cocktail) at 4 °C for 30 min. Samples were cleared at 20,000 *rcf* for 15 min. Histones were extracted from the insoluble fraction using 0.2 M sulphuric acid overnight at 4 °C. Approximately 30 μg protein were resolved on using SDS/PAGE and nitrocellulose membranes were probed with the appropriate antibodies (Supplementary Table 1). The Odyssey Infrared Imaging System was used to quantify protein signal using the appropriate IR-Dye conjugated secondary antibodies (LI-COR Biosciences).

### Chromatin immunoprecipitation (ChIP)

*Hdac1/2* DKO ES cells were processed for native ChIP-seq as previously described^44,45^. Briefly, cell pellets were swollen in 0.3 M Sucrose, 60 mM KCl, 15 mM NaCl, 5 mM MgCl_2_, 0.1 mM EGTA, 15 mM Tris-HCl, pH 7.5 and EDTA-free protease inhibitors (Sigma-Aldrich, UK). Nuclei were extracted using an equal volume of 0.3 M sucrose, 60 mM KCl, 15 mM NaCl, 5 mM MgCl_2_, 0.1 mM EGTA, 15 mM Tris-HCl, pH 7.5, 0.4% NP-40, 0.5 mM DTT and protease inhibitors at 4 °C for 10 min. The cell suspension was applied to a sucrose cushion containing 1.2 M sucrose, 60 mM KCl, 15 mM NaCl, 5 mM MgCl_2_, 0.1 mM EGTA, 15 mM Tris-HC pH 7.5 and centrifuge at 1500 *rcf* for 30 min at 4 °C. Nuclei were resuspended in MNase Digestion Buffer containing 0.32 M sucrose, 50 mM Tris-HCl, pH 7.5, 4 mM MgCl_2_, 1 mM CaCl2 and protease inhibitors. Chromatin was then digested using 2UN/ml-1 Micrococcal nuclease (MNase) for 10–12 min at 37 °C on a thermal shaker. The reaction was stopped with 5 μM EDTA/EGTA and cleared at 12,000 *rcf* for 10 min at 4 °C. The supernatant (S1 fraction) was stored a 4 °C and the pellet was dialysed with 1 mM Tris-HCl, pH 7.5 and 0.2 mM EDTA overnight at 4 °C. The dialysed pellet (S2 fraction) was cleared at 20,000 *rcf* for 10 min at 4 °C and combined with S1 fraction for immunoprecipitation. Chromatin was immunoprecipitated using 8 μg H3K18ac (Catalog No: 39755; Active Motif) or 8 μg H3K18cr (PTM-Biolabs 517) conjugated to 50 μL Dynabeads Protein G overnight at 4 °C. Bead were washed for 5 min in wash buffer A (50 mM Tris-HCl, pH 7.5, 10 mM EDTA, 75 mM NaCl), wash buffer B (50 mM Tris-HCl, pH 7.5, 10 mM EDTA, 125 mM NaCl), and wash buffer C (50 mM Tris-HCl, pH 7.5, 10 mM EDTA, 175 mM NaCl). DNA was purified using IPure kit v2 (Diagenode, Belgium) according to the manufacturer’s instructions.

### ChIP-seq data analysis

For next-generation sequencing, automated library preparation were prepared from 10 ng of ChIP, and input DNA using the Apollo prep system (Wafergen, PrepX ILMN 32i, 96 sample kit) and standard Illumina multiplexing adapters following manufacturer’s protocol up to pre-PCR amplification. Libraries were PCR amplified (18 cycles) on a Tetrad (Bio-Rad) using the NEBNext High-Fidelity 2X PCR Master Mix (NEB) and in-house (Oxford Genomics Centre) dual indexing primers^46^. Paired end sequencing was performed using a HiSeq4000 75 bp platform (Illumina, HiSeq3000/4000 PE Cluster Kit and 150 cycle SBS Kit), generating a raw count >28 million reads per sample. Data from these experiments has been deposited at the Gene Expression Omnibus (GEO) database, accession number GSE116019.

Primary analysis pipeline and data QC included alignment of reads to the mm9 genome assembly using BWA-mem. Peaks were called using MACS (version 2.1.1.20160309) with default parameters. Deeptools package (version 2.5.4^47^) was used to producing heatmaps, and profile plots. Meta-analysis of H3K18ac and H3K18cr levels at bivalent genes (GSE12241^36^) and genes upregulated following the deletion of HDAC1/2 (DKO; GSE52134^29^) was performed using Deeptools. Briefly, bamCompare was used to produce bigwig files of normalized read depth (H3K18ac/Input and H3K18cr/Input). Gene lists were used as a reference along with the normalized bigwig files to produce matrix files of scores around genome features of interest (computeMatrix). Matrix scores at respective transcription start sites (TSS; 500 bp) were plotted in Prizm (GraphPad version 7) and statistically significant differences were derived from a Mann–Whitney U test. Similarly, the top 10% matrix scores for either H3K18cr or H3K18ac at TSS (5000 bp) were used to extract genes lists and gene ontology (GO) analysis was carried out using the Database for Annotation, Visualization, and Integrated Discovery (DAVID), version 6.8^48^.

For HDAC1/2 ChIP-Seq data sets (GSE27844^38^) read trimming was performed using Trimmomatic (version 0.36). Bowtie2 (version 2.2.9) was used to map sequence reads to mouse reference genome mm9 and Samtools (version 1.3.2) for sam/bam file processing. Deeptools package (version 2.5.4) were used for producing heatmaps.

## Electronic supplementary material

Supplemental Information
