# Supplementary material for: Histone deacetylase (HDAC) 1 and 2 complexes regulate both histone acetylation and crotonylation in vivo
Source: Sci Rep. 2018 Oct 2;8:14690. doi: 10.1038/s41598-018-32927-9 (PMC6168483; doi:10.1038/s41598-018-32927-9)

Histone deacetylase (HDAC) 1 and 2 complexes regulate both histone acetylation and crotonylation *in vivo*.

Kelly RDW[1], Chandru A[1], Watson PJ[2], Song Y[2], Blades M[3], Robertson NS[5], Jamieson A[4], Schwabe JWR[2] and Cowley SM[1,*]

[1] Department of Molecular and Cell biology, Henry Wellcome Building, University of Leicester, Leicester, LE1 9HN, UK

[2] Institute of Structural and Chemical biology, Henry Wellcome Building, Department of Molecular and Cell biology, University of Leicester, Leicester, LE1 9HN, UK

[3] Bioinformatics and Biostatistics Analysis Support Hub (B/BASH), University of Leicester, Leicester, LE1 7RH, UK

[4] School of Chemistry, Joseph Black Building, University Avenue, University of Glasgow, Glasgow G12 8QQ

[5] Department of Chemistry, University of Cambridge, Cambridge. CB2 1GA, UK

*Corresponding author smc57@le.ac.uk

Supplemental Information

Supplementary Figures 1-4 page 1-9

Supplemental Table 1 page 10

Supplemental Methods:

1. Synthesis of Boc-Lys(Cr)-AMC substrate page 11
2. Peptide Synthesis and characterization page 12-13
3. RP-HPLC and MS data page 14-19

Supplemental Figure 1

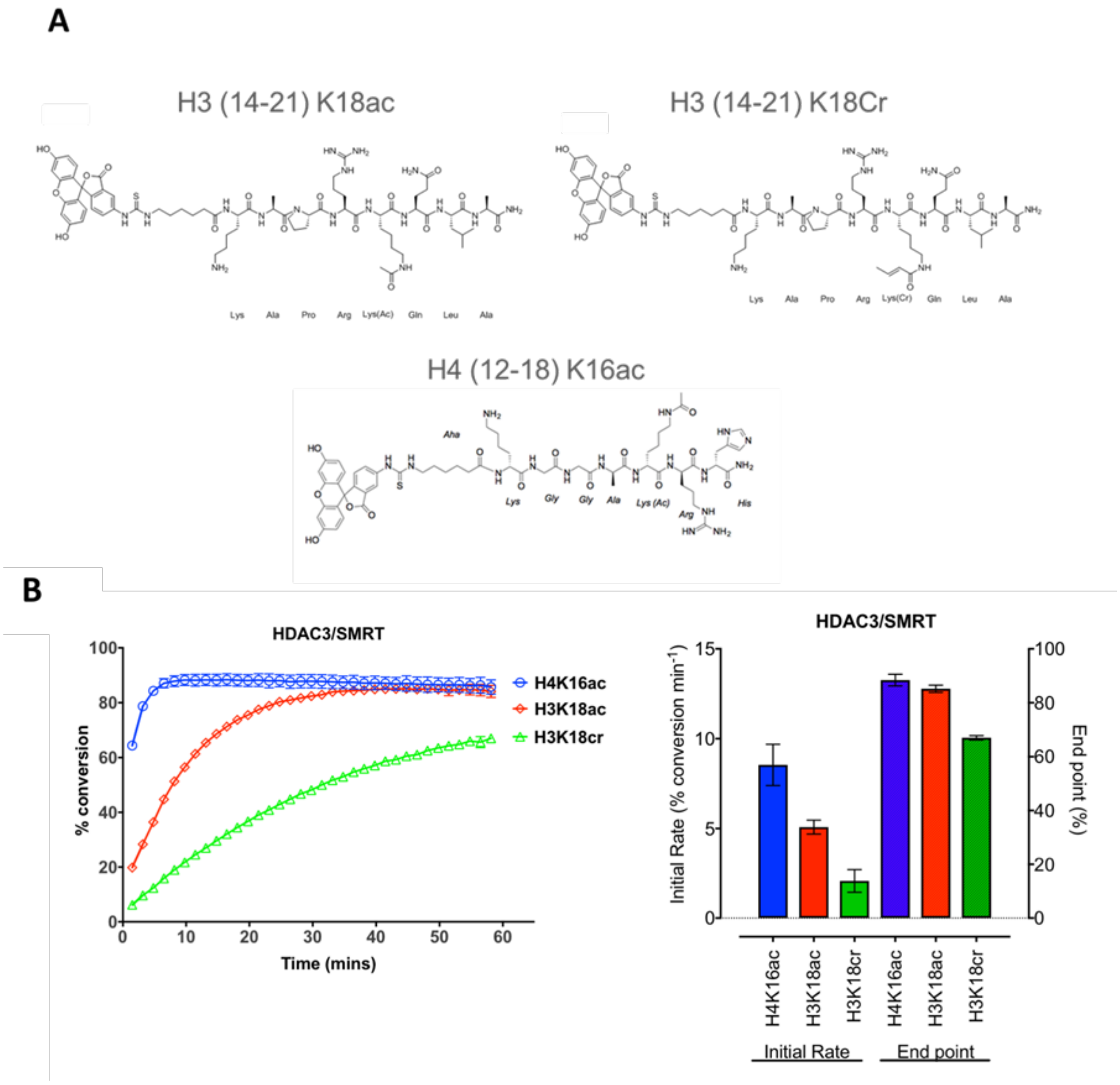

Supplementary Figure 1: Peptides used in Caliper assays and Deacylase activity of the HDAC3/SMRT complex. (A) Fluorescein-labelled peptides based on histone H3 (residues 14-21) and H4 (residues 12-18) tails used for Caliper assays in Fig. 1. The H4 peptide was acetylated at Lys16 and H3 acetylated and crotonylated at Lys18, as indicated. These peptides we incubated with recombinant HDAC3/SMRT/TBL1/GPS2 quaternary complex (B). Deacetylase and decrotonylase activities were measured using a Caliper assay. Graphs show the average percentage conversion (mean ± SD; n=2), initial rates (mean ± SD; n=2), and percentage conversion at the end point of the assay (mean ± SD; n=2).

Supplemental Figure 2

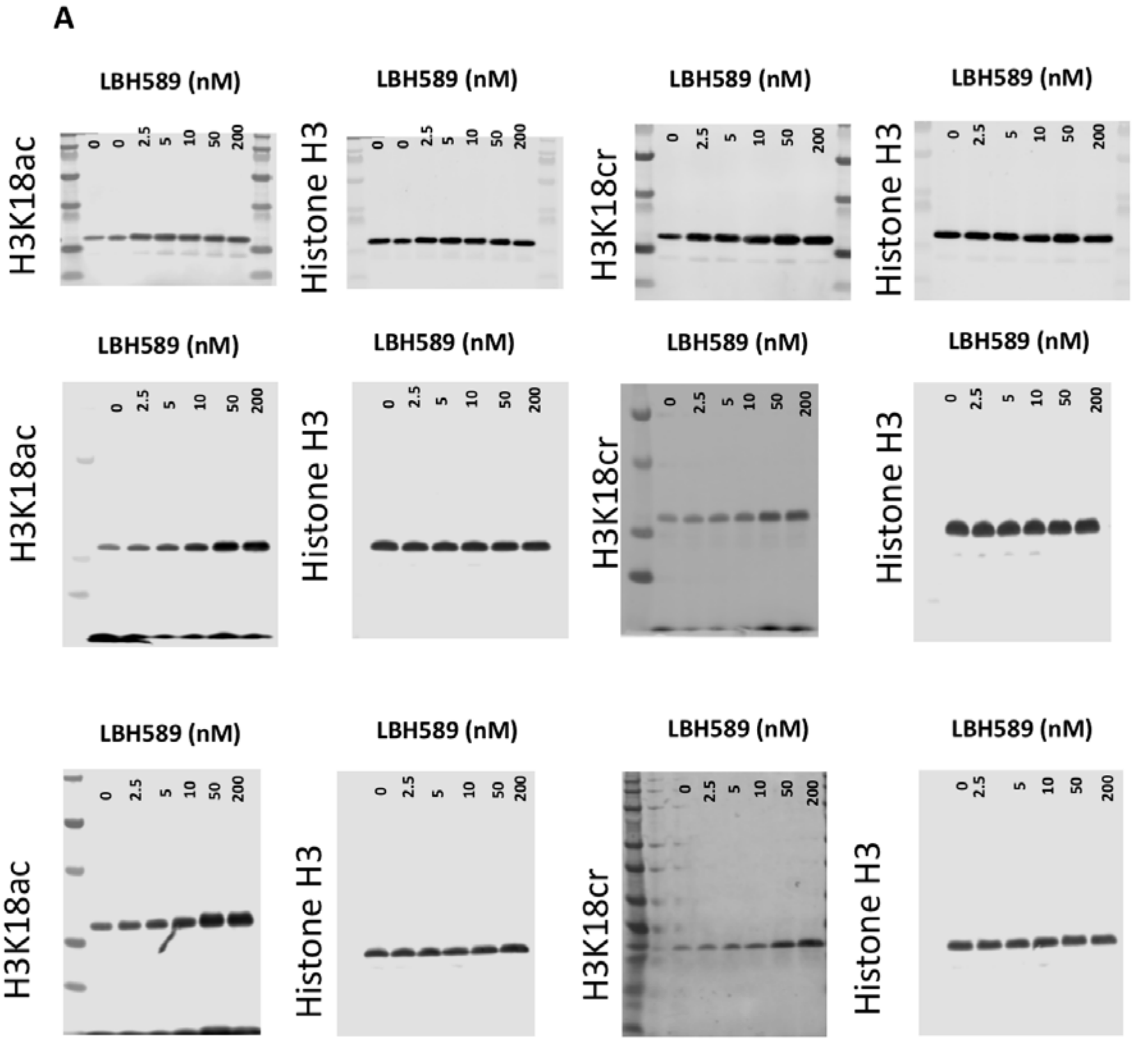

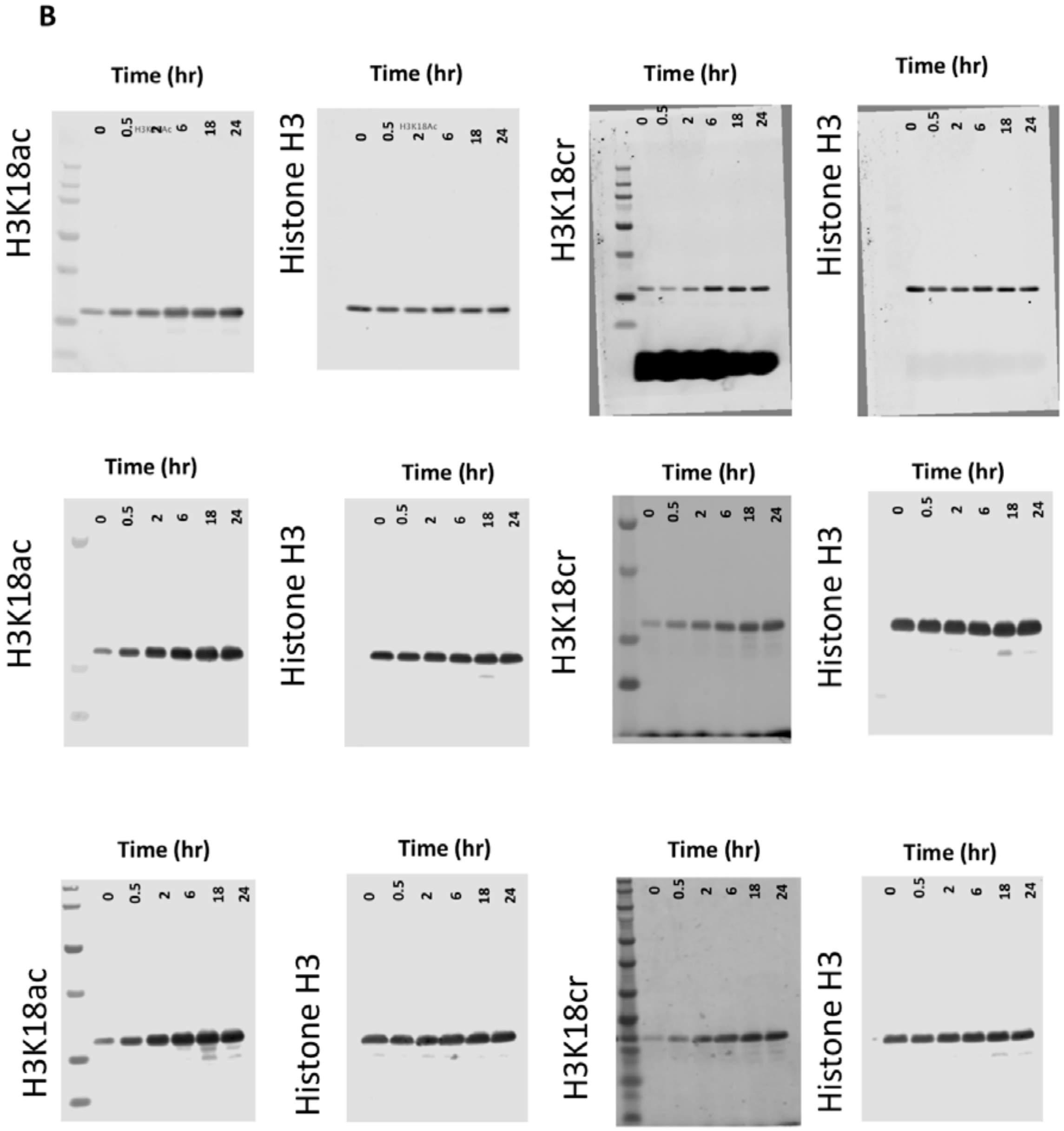
B
Time (hr)
0
0.5
2
6
18
24
H3K18ac
Histone H3
H3K18cr

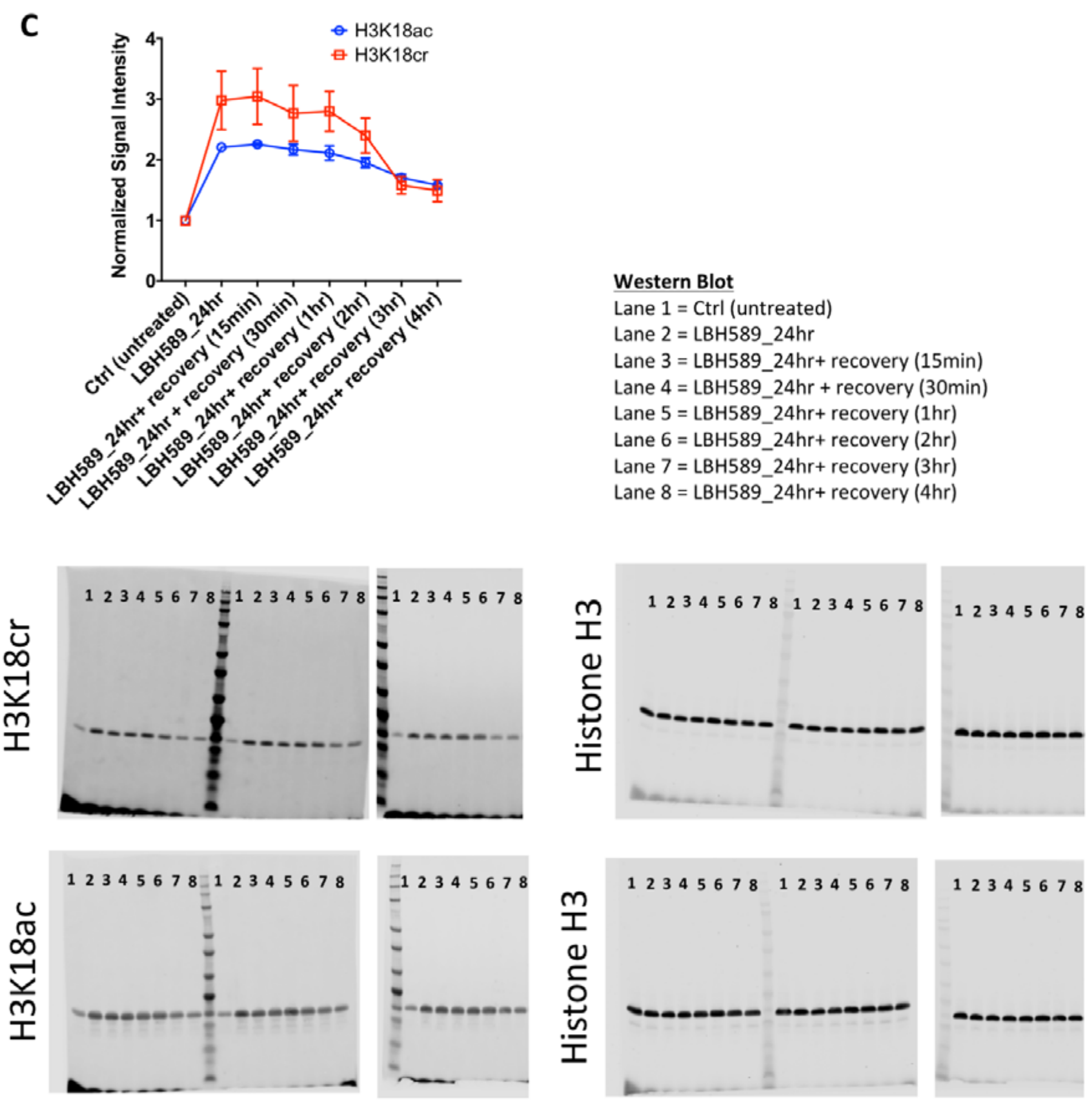

Supplemental Figure 2: HDAC inhibition increases both H3K18ac and H3K18cr in a dose-dependent manner. (A) Uncropped scans of western blot gels as depicted in Figure 2C. (B) Uncropped scans of western blot gels as depicted in Figure 2D. (C) Quantitative western blot data for H3K18ac and H3K18cr levels following washing out LBH589 (30nM) after a 24hr treatment. Cells were cultured in the absence LBH589 for up to 4hrs. Graph show the average normalized signal intensity (mean ±SEM; n=3) relative to Histone H3. Uncropped scans of western blot gels shown below graph.

Supplemental Figure 3

A

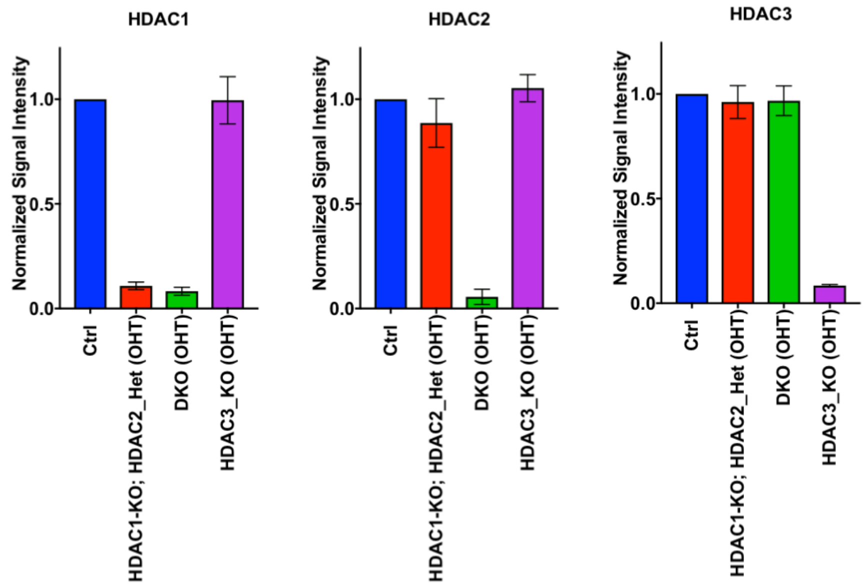

Western Blotting

Lane 1 = HDAC1-KO; HDAC2_Het (CTRL)

Lane 2 = HDAC1-KO; HDAC2_Het (OHT_Day3)

Lane 3 = DKO (CTRL)

Lane 4 = DKO (OHT_Day3)

Lane 5 = HDAC3-KO (CTRL)

Lane 6 = HDAC3-KO (OHT_Day3)

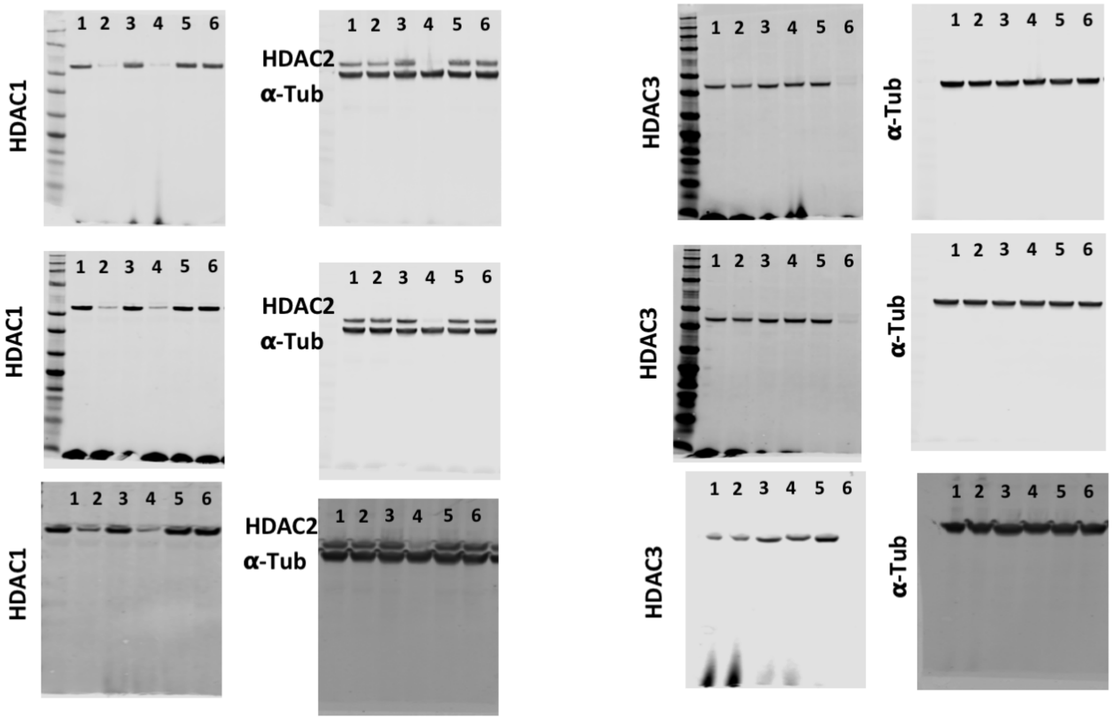

B

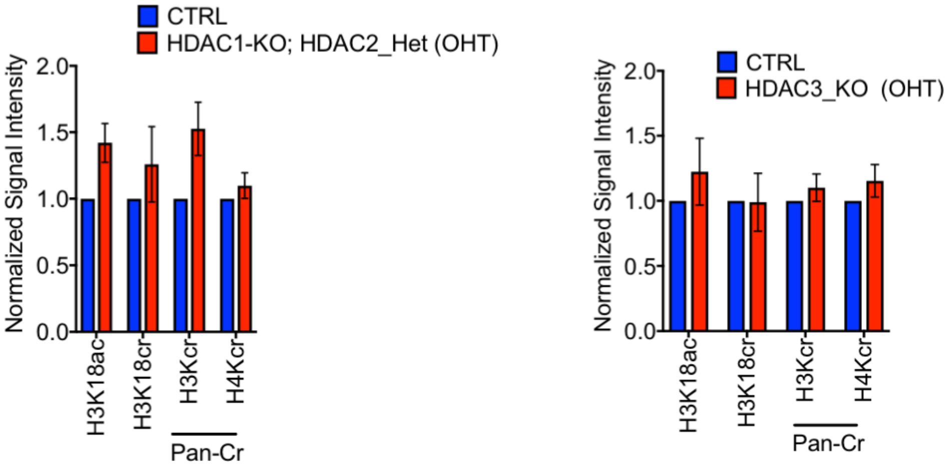

Western Blotting

Lane 1 = HDAC1-KO; HDAC2_Het (CTRL)
Lane 2 = HDAC1-KO; HDAC2_Het (OHT_Day3)
Lane 3 = DKO (CTRL)
Lane 4 = DKO (OHT_Day3)
Lane 5 = HDAC3-KO (CTRL)
Lane 6 = HDAC3-KO (OHT_Day3)

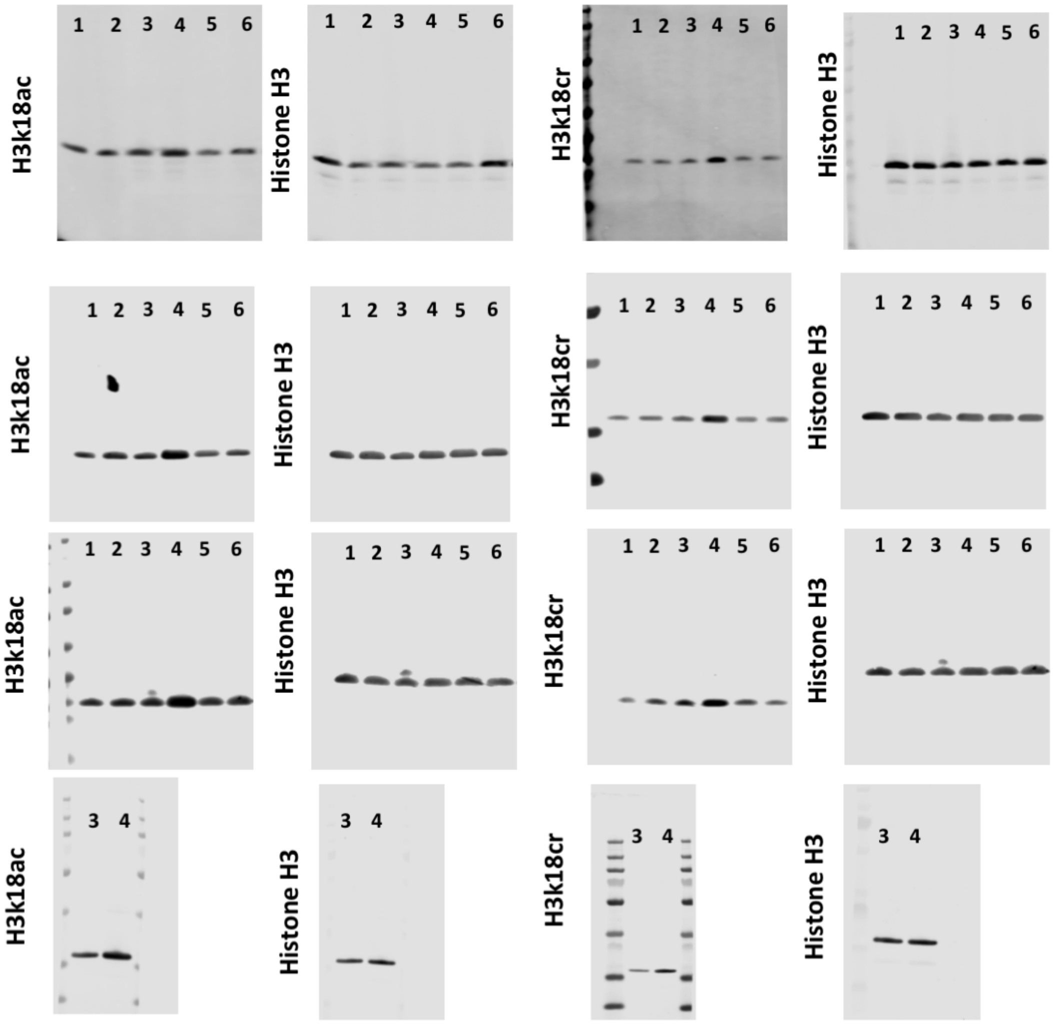

C

Western Blotting

Lane 1= HDAC1-KO; HDAC2_Het (CTRL)
Lane 2= HDAC1-KO; HDAC2_Het (OHT_Day3)
Lane 3 = DKO (CTRL)
Lane 4 = DKO (OHT_Day3)
Lane 5 = HDAC3-KO (CTRL)
Lane 6 = HDAC3-KO (OHT_Day3)

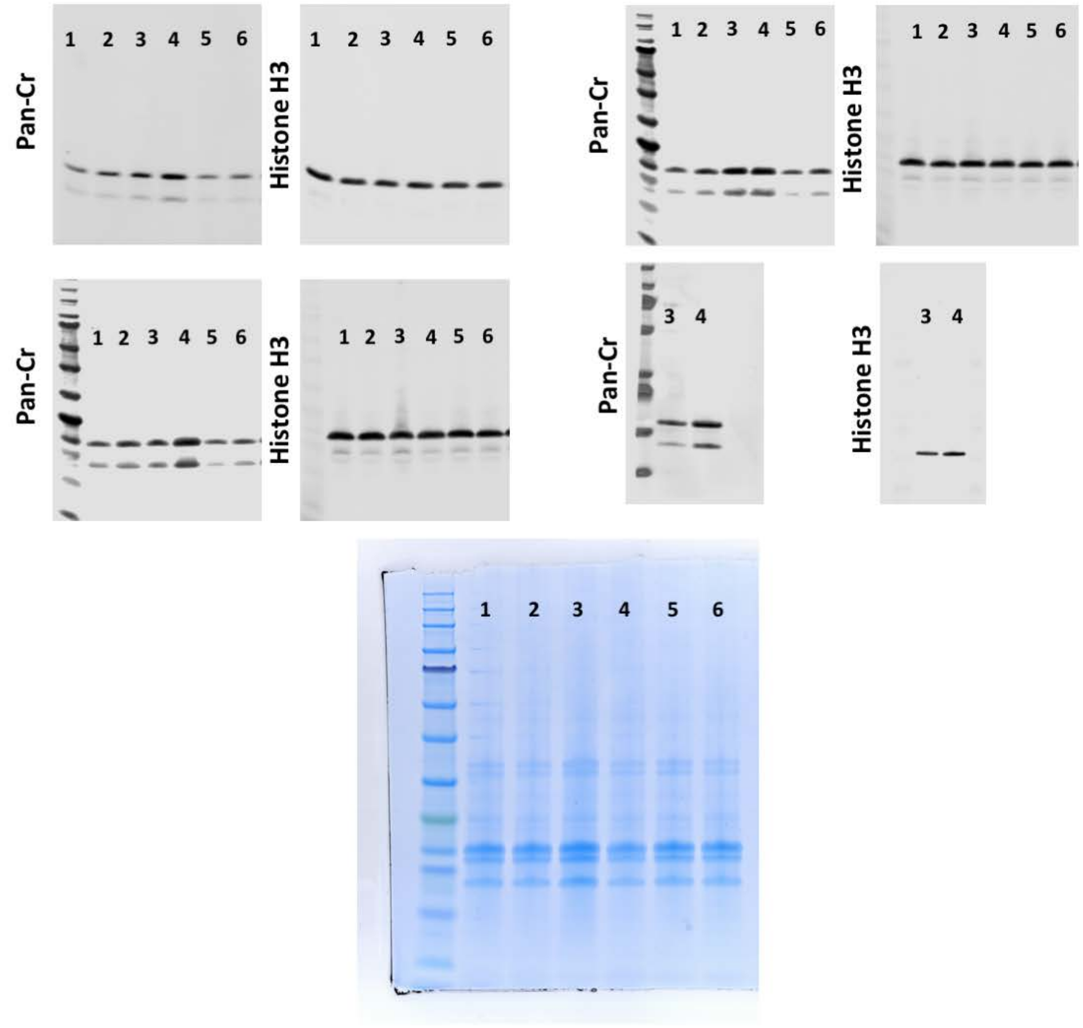

Supplementary Figure 3: HDAC1/2 are the dominant histone decrotonylase in ES cells. Uncropped scanned western blot gels as depicted in Figure 3B. Quantitative western blot data for (A) HDAC1, 2 and 3; (B) H3K18ac and H3K18cr (acid extracted histones); (C) Pan-Cr (acid extracted histones) for the indicated cells lines in Ctrl (untreated) and knock-out (KO) ES cells treated with 4-hydroxytamoxifen (OHT) at day 3. Poly-acrylamide gel stained with Instant Blue protein (C) to visualize individual histones. All graphs show the average normalized signal intensity (mean ±SEM; n≥3) relative to Histone H3.

Supplemental Figure 4

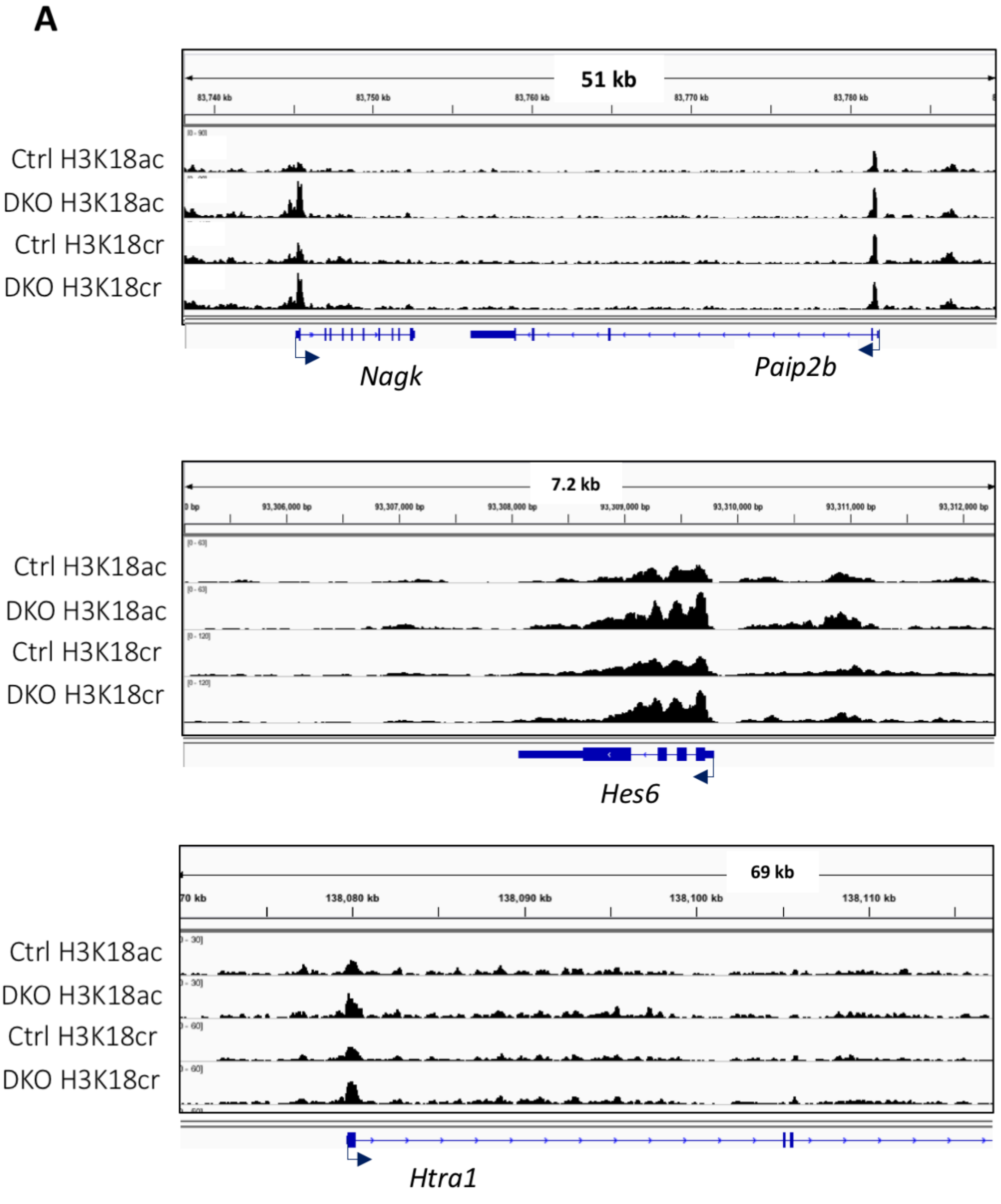

Supplementary Figure 4: H3K18ac and H3K18cr levels around TSS correlate with increase gene expression following the loss of HDAC1/2 in ES cells. Images show IGV enrichment tracks of H3K18ac and H3K18cr at the four representative genes: *Nagk, Paip2b, Hes6* and *Htra1*. The gene structure and dimensions are indicated in each panel.

Supplementary Table 1: list of antibodies used in this study

Antibody Manufacturer and	Catalog/Product Number	Dilutions Used
Anti-HDAC1 antibody [EPR460(2)] Rabbit mAb	AbcamCat: Ab 109411	WB 1:1000
Anti-HDAC2, clone 3F3	Millipore<br>Catalog number: 05-814	WB 1:1000 IP: 1:20
Anti-HDAC3	Abcam – ab32369	WB 1:2000
Anti α-Tubulin	Sigma – T5168	WB 1:15000
Anti-KDM1 / LSD1 antibody [EPR6825]	Abcam<br>Cat: Ab129195	WB: 1:10,000<br>IP: 1:4
Anti-acetyl-Histone H3 (Lys18) Rabbit pAb	Active Motif Catalog No: 39755	WB 1:10,000; 8μg ChIP-seq
Anti Crotonyl-Histone H3 Lys18 Rabbit pAb	PTM Biolabs LLC<br>Item code: PTM-517	WB 1:2000; 8μg ChIP-seq
Anti Pan anti-crotonyllysine Rabbit pAb	PTM Biolabs LLC Item code: PTM-501	WB 1:1000
Anti-Histone H3, clone 6.6.2	Millipore Cat. # 05-499	WB 1:2000

Supplementary Methods

1. Synthesis of Boc-Lys(Cr)-AMC substrate

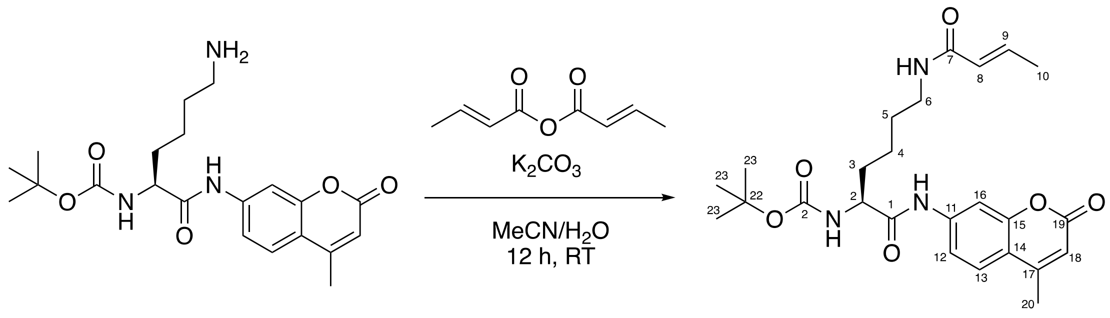

Boc-Lys-AMC (50 mg, 0.12 mmol, 1 equiv.) was dissolved in *N,N'*-dimethylformamide (88 μL, 1.36 M) then diluted with acetonitrile (882 μL, 0.136 M) before the addition of potassium carbonate (34 mg, 0.25 mmol, 2 equiv.) and crotonic anhydride (20 μL, 0.14 mmol, 1.1 equiv.). The solution was stirred at room temperature for 12 h. The solvent was removed *in vacuo* then the solid resuspended in water (5 mL) and extracted with dichloromethane (3 x 5 mL). The combined organic layers were washed sequentially with 5% lithium chloride solution (3 x 15 mL) and brine (3 x 15 mL), then dried ($MgSO_4$) before the solvent was removed *in vacuo* to give Boc-Lys(Cr)-AMC (33 mg, 58%) as a white powder.

^{1}H NMR (500 MHz, $CDCl_3$) δ ppm 9.61 (1H, br s, N*H*$_{AMC}$), 7.69 (1H, s, H-16) 7.44 (1H, m, H-13), 7.39 (1H, m, H-12), 6.81 (1H, m, H-9), 6.13 (2H, br s, NH_{crot}, H-18), 5.82 (1H, d, *J*=15.3 Hz, H-8), 5.68 (1H, d, *J*=6.6 Hz, NHBoc), 4.32 (1H, m, H-2), 3.31 (2H, m, H-6), 2.37 (3H, s, CH_3-20), 1.94 (1H, m, H-3), 1.82 (3H, d, *J*=6.2 Hz, CH_3-10), 1.67 – 1.78 (1H, m, H-3'), 1.51 – 1.64 (2H, m, CH_2-5), 1.37 – 1.51 (11H, m, $(CH_3)_3$-23, CH_2-4). **^{13}H NMR** (125 MHz, $CDCl_3$) δ ppm 171.7 (C=O, C-1), 1.66.4 (C=O, C-7), 161.2 (C=O, C19), 156.4 (C=O, C-21), 154.0 (qC-15), 152.5 (qC-17), 141.7 (qC-11), 140.0 (CH-9), 125.0 (CH-13), 125.0 (CH-8), 115.7 (qC-14), 115.6 (CH-12), 113.0 (CH-18), 107.1 (CH-16), 80.3 (qC-22), 55.1 (CH-2), 38.4 (CH_2-6), 31.6 (CH_2-6), 28.9 (CH_2-5), 28.3 (CH_3-23), 22.6 (CH_2-4), 18.5 (CH_3-20), 17.6 (CH_3-10). **MS** (ESI+) *m/z* 510 $[M+K]^+$.

2. Peptide Synthesis and Characterization

Peptides were synthesised on a Biotage Initiator + Alstra microwave assisted peptide synthesiser using an Fmoc/^{t}Bu protecting group strategy and standard methods on a 0.1 mmol synthetic scale using Rink Amide ChemMatrix Resin (0.43 mmol/g loading).

Deprotection reactions were performed using 20% piperidine in DMF (v/v) containing 0.1 M oxyma pure, at 75 °C (mW) for 30 sec, followed by a second deprotection using fresh solution at 75 °C for 3 min.

Fmoc-protected amino acids were coupled using HCTU activator (0.25 M solution in DMF, 4 equiv.) and *N*,*N*-diisopropylethylamine (DIEA) activator base (1M solution in NMP, 8 equiv.) at 75 °C for 10 min. Double coupling of Fmoc-Arg(Pbf)-OH was carried out at room temperature for 25 min, then heated to 75 °C for 5 min, drained and a second coupling step carried out at 75 °C for 5 min. Fmoc-His(Trt)-OH was coupled at 50 °C for 10 min.

Fluorescein isothiocyanate (2 equiv.) was coupled to the peptide N-terminal in DMF (0.1M) for 3 h at ambient temperature.

Peptide cleavage was performed using a cleavage cocktail of 95% Trifluoroacetic acid (TFA), 2.5% Triisopropylsilane (TIS), 2.5% water and the resin stirred for 3 h at ambient temperature. The cleavage cocktail was evaporated using a stream of nitrogen and the peptide precipitated from solution with ice cold Et_2O. The tube was spun in a centrifuge (5000 rpm) and the peptide recovered as a pellet following decanting of the ether solution.

Peptides were then purified by reverse-phase HPLC (RP-HPLC) with a Gemini™ 5 µm C18 110 Å column (Phenomenex® Inc., Torrance, California, 250 mm × 21 mm, 5 µm, C18) using a specified linear gradient of acetonitrile (0.1% TFA) in water (0.1% TFA), with a flow rate of 10.6 mL and collected fractions were lyophilised on a Christ Alpha 2-4 LO plus freeze dryer.

Pure peptides were analysed by RP-HPLC using a Phenomenex, Aeris, 5 µm, peptide XB-C18, 150 x 4.6 mm column at a flow rate of 1 mL/min. RP-HPLC gradients were run using a solvent system consisting of solution A (5% MeCN in H_2O + 0.1% TFA) and B (5% H_2O in MeCN + 0.1% TFA). Analytical RP-HPLC data is reported as column retention time (t_R) in minutes (min). High resolution mass spectrometry (HRMS) data are reported as mass to charge ratio (m/z) = observed / MW.

FITC-Ahx-Lys-Ala-Pro-Arg-Lys(Ac)-Asn-Leu-Ala-NH_2 (FITC Ahx H3(14-21)K18Ac): 67.1 mg, 46% yield (>99% purity); HRMS (m/z) [M+H]+ calcd for $C_{69}H_{100}N_{17}O_{16}S$, 1454.7355; found 1454.7323 (Δ 4.7 ppm); analytical HPLC 5-100% acetonitrile/water (0.1% trifluoroacetic acid), 15 min gradient, T_R 18.720 min; 5-100% acetonitrile/water (0.1% trifluoroacetic acid), 30 min gradient, T_R 23.267 min.

FITC-Ahx-Lys-Ala-Pro-Arg-Lys(Cr)-Asn-Leu-Ala-NH_2 (FITC Ahx H3(14-21)K18Cr): 104.9 mg, 71% yield (>99% purity); HRMS (m/z) [M+H]+ calcd for $C_{71}H_{102}N_{17}O_{16}S$, 1480.7411; found 1480.7441 (Δ 2.0 ppm); analytical HPLC 5-100% acetonitrile/water (0.1% trifluoroacetic acid), 15 min gradient, T_R 19.687 min; 5-100% acetonitrile/water (0.1% trifluoroacetic acid), 30 min gradient, T_R 24.073 min.

FITC-Ahx-Lys-Gly-Gly-Ala-Lys(Ac)-Arg-His-NH_2 (FITC Ahx H3(12-18)K16Ac): 42 mg, 33% yield (>99% purity); HRMS (m/z) [M+H]+ calcd for $C_{60}H_{82}N_{17}O_{14}S$, 1296.5948; found 1296.5977 (Δ 2.2 ppm); analytical HPLC 5-100% acetonitrile/water (0.1% trifluoroacetic acid), 15 min gradient, T_R 13.757 min; 5-100% acetonitrile/water (0.1% trifluoroacetic acid), 30 min gradient, T_R 18.443 min.

3. RP-HPLC and MS data

FITC-Ahx-Lys-Ala-Pro-Arg-Lys(Ac)-Asn-Leu-Ala-NH_2 (FITC Ahx H3(14-21)K18Ac)

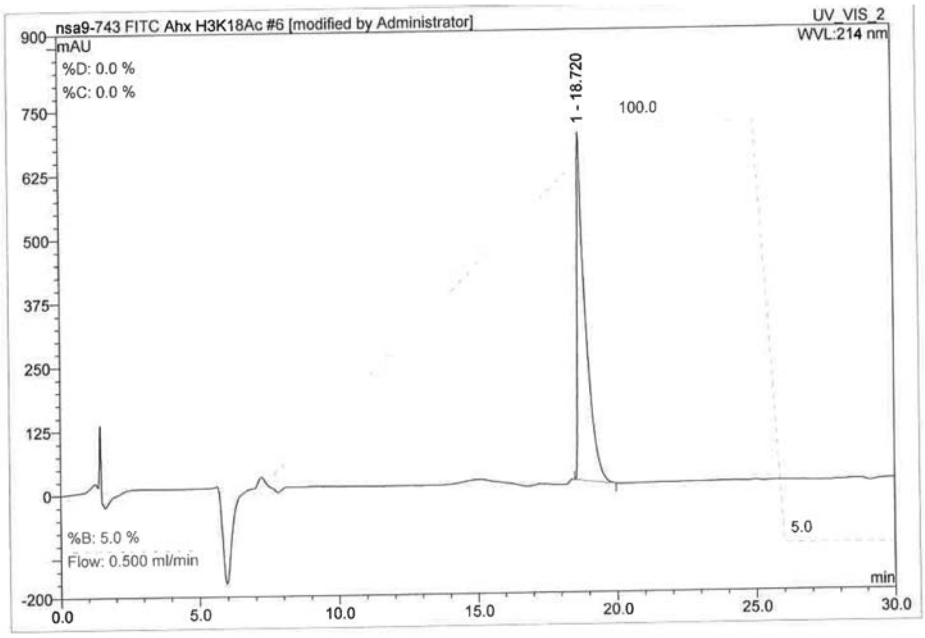

Analytical HPLC 5-100% acetonitrile/water (0.1% trifluoroacetic acid), 15 min gradient

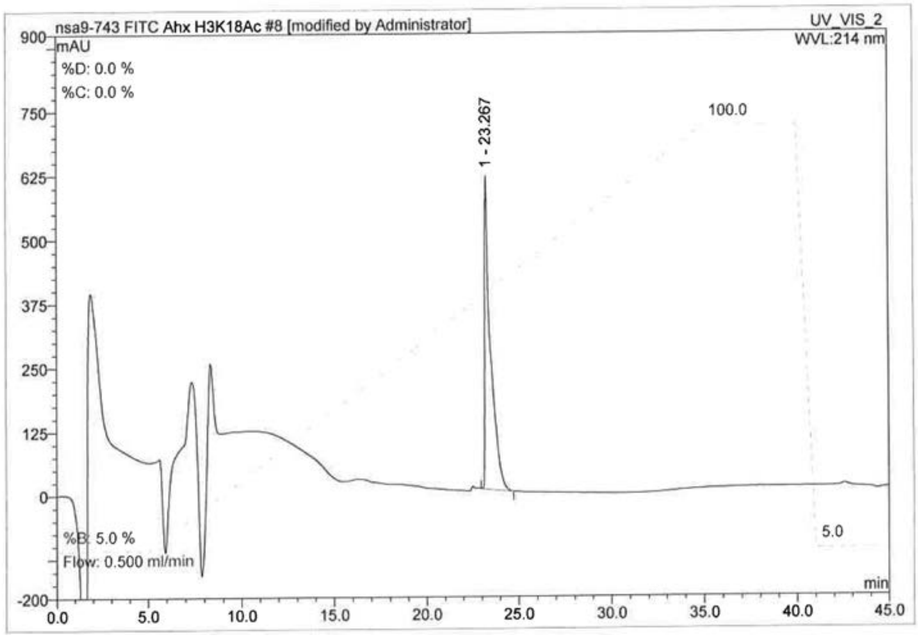

Analytical HPLC 5-100% acetonitrile/water (0.1% trifluoroacetic acid), 30 min gradient

ESI Mass Spectrometry - Chemical Formula: $C_{69}H_{100}N_{17}O_{16}S$; MW 1454

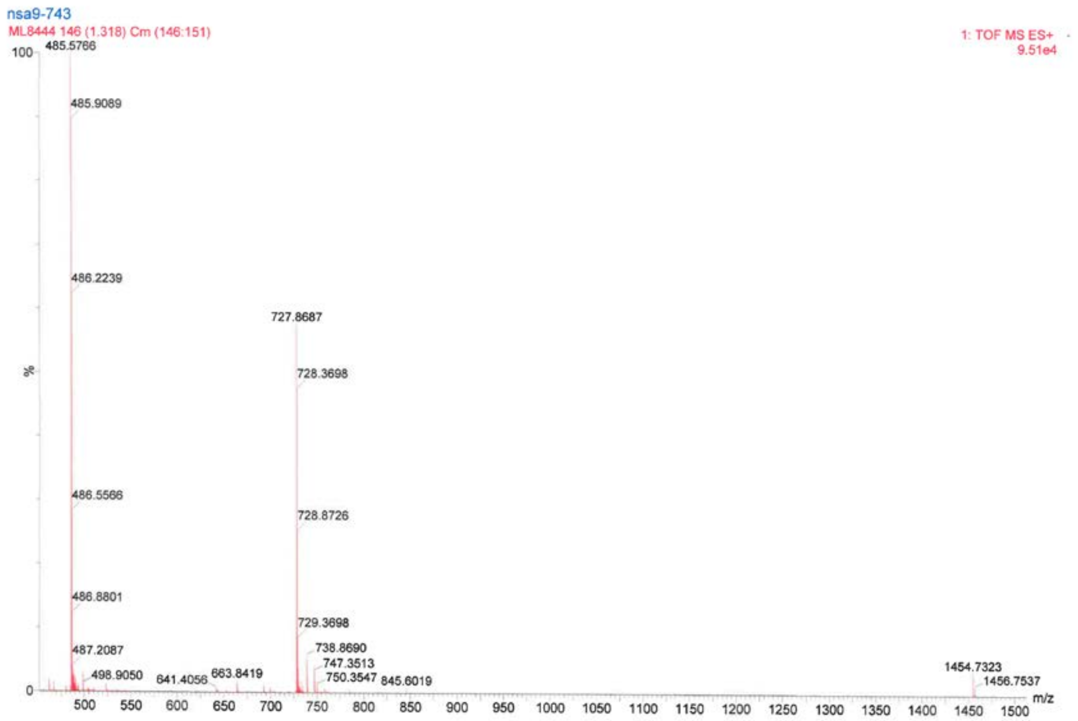

FITC-Ahx-Lys-Ala-Pro-Arg-Lys(Cr)-Asn-Leu-Ala-NH_2 (FITC Ahx H3(14-21)K18Cr)

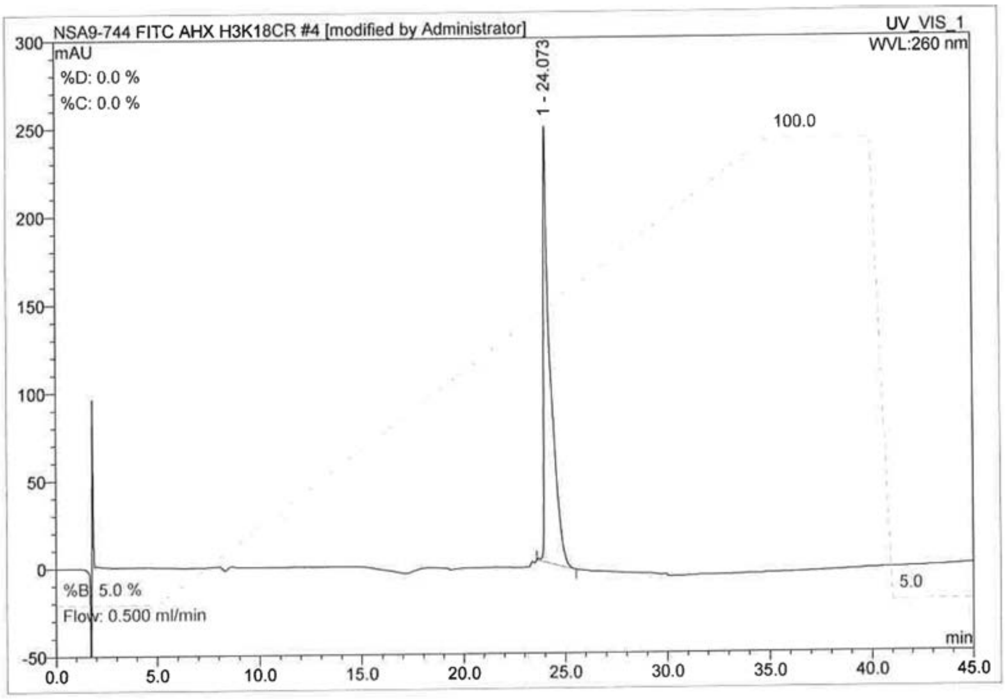

Analytical HPLC 5-100% acetonitrile/water (0.1% trifluoroacetic acid), 15 min gradient

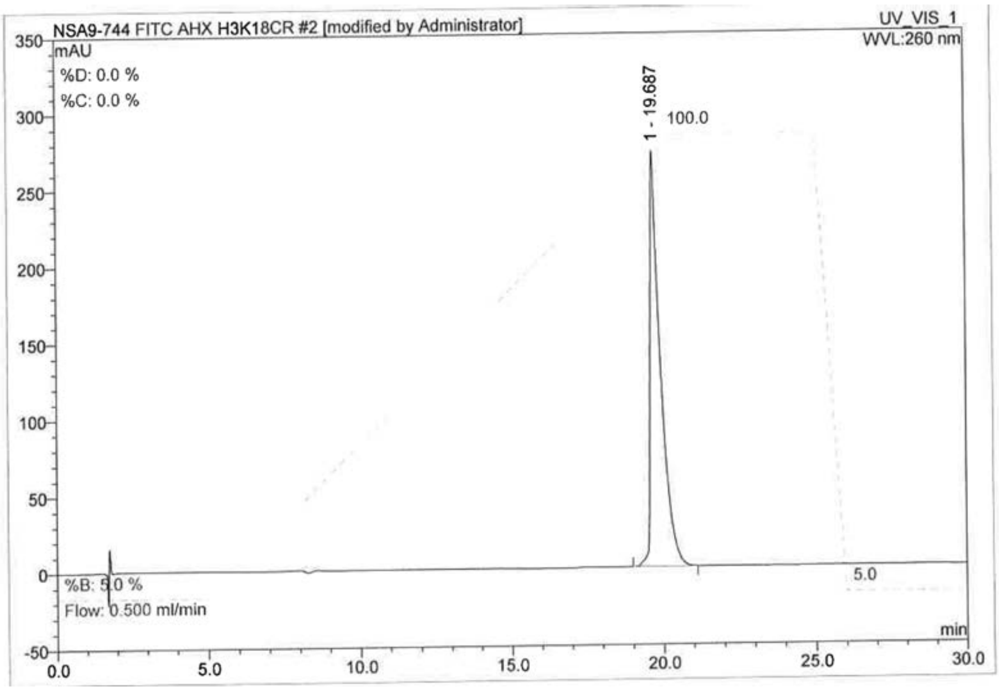

Analytical HPLC 5-100% acetonitrile/water (0.1% trifluoroacetic acid), 30 min gradient

ESI Mass Spectrometry - Chemical Formula: $C_{71}H_{102}N_{17}O_{16}S$; MW 1480

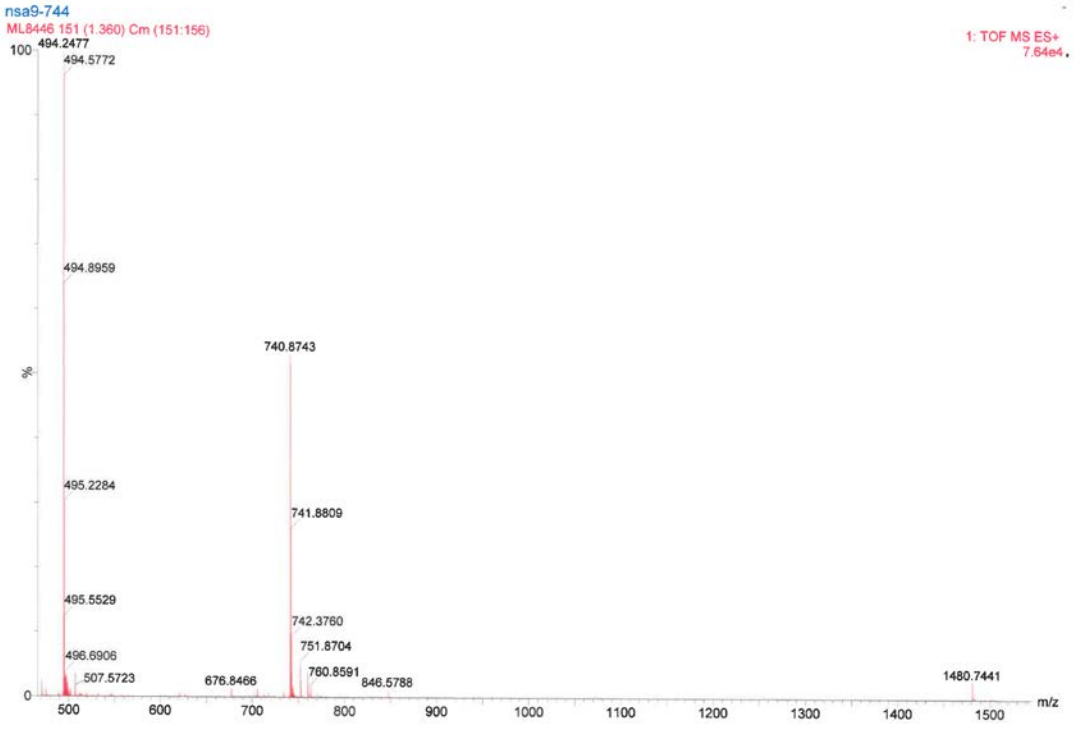

FITC-Ahx-Lys-Gly-Gly-Ala-Lys(Ac)-Arg-His-NH_2 (FITC Ahx H3(12-18)K16Ac)

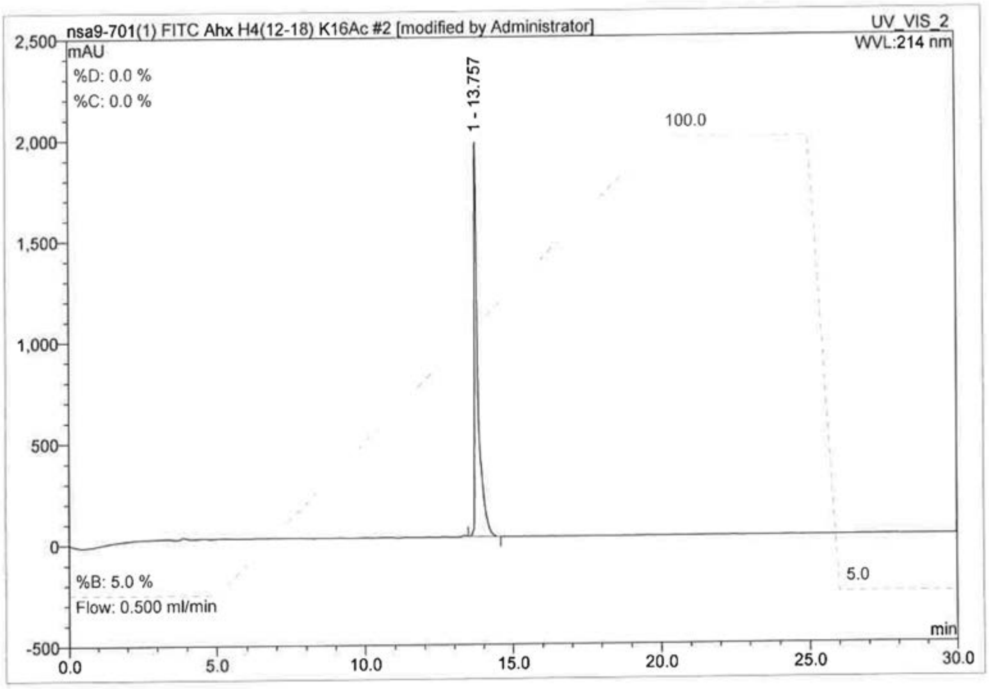

Analytical HPLC 5-100% acetonitrile/water (0.1% trifluoroacetic acid), 15 min gradient

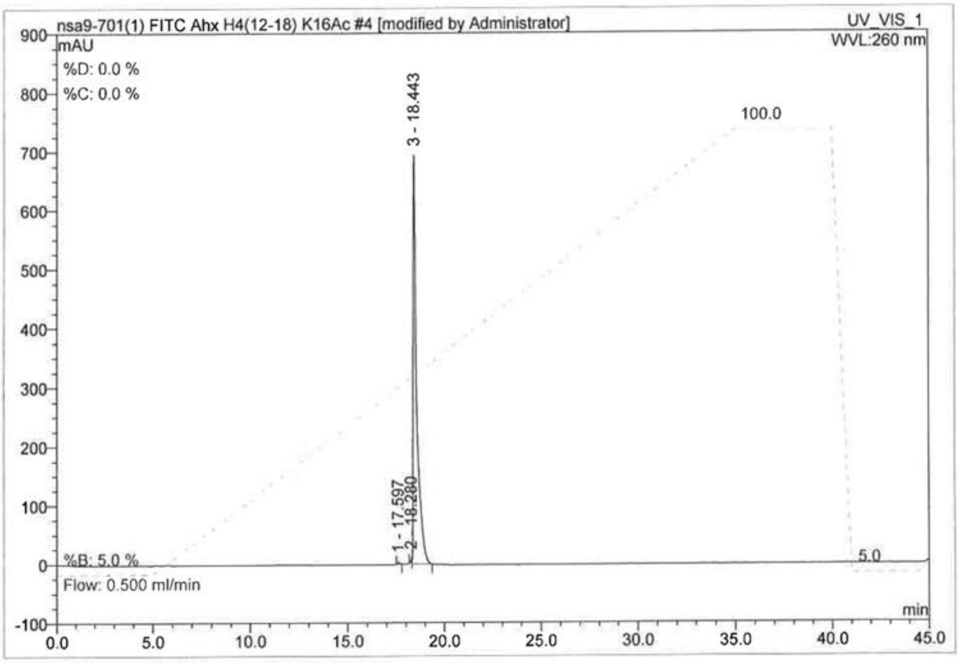

Analytical HPLC 5-100% acetonitrile/water (0.1% trifluoroacetic acid), 30 min gradient

ESI Mass Spectrometry - Chemical Formula: $C_{69}H_{100}N_{17}O_{16}S$; MW 1296

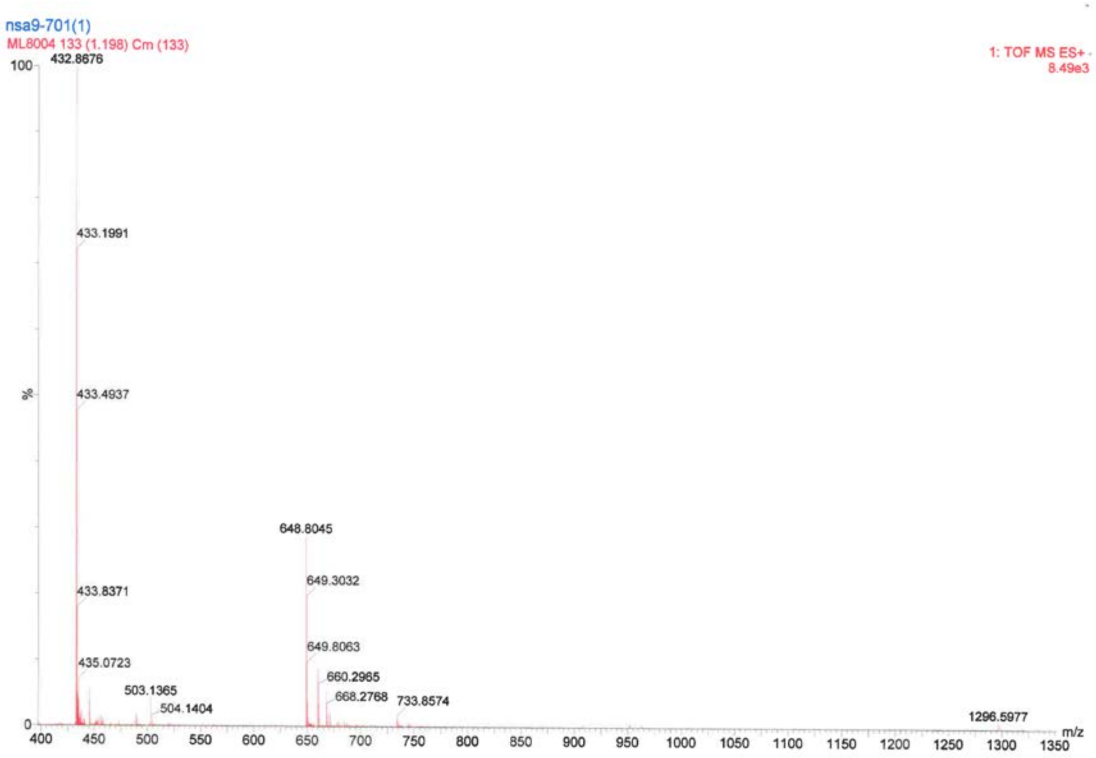

Supplement: Supplementary file 1 — Supplemental Information [file 41598_2018_32927_MOESM1_ESM.pdf]
